# Glutathione Pulse Therapy: Promote Spatiotemporal Delivery of Reduction‐Sensitive Nanoparticles at the “Cellular Level” and Synergize PD‐1 Blockade Therapy

**DOI:** 10.1002/advs.202202744

**Published:** 2022-07-27

**Authors:** Songtao Dong, Yuan Zhang, Xiangnan Guo, Chuang Zhang, Zhaomeng Wang, Jiang Yu, Yubo Liu, Chang Li, Yuting Hu, Bingjun Sun, Mengchi Sun, Haotian Zhang, Defang Ouyang, Zhonggui He, Yongjun Wang

**Affiliations:** ^1^ Department of Pharmaceutics Wuya College of Innovation Shenyang Pharmaceutical University Shenyang 110016 China; ^2^ School of Pharmacy China Medical University Shenyang 110122 China; ^3^ State Key Laboratory of Quality Research in Chinese Medicine Institute of Chinese Medical Sciences (ICMS) University of Macau Macau 999078 China

**Keywords:** glutathione, MALDI‐MSI, nanoparticles, PD‐1, spatiotemporal delivery

## Abstract

Spatiotemporal delivery of nanoparticles (NPs) at the “cellular level” is critical for nanomedicine, which is expected to deliver as much cytotoxic drug into cancer cells as possible when NPs accumulate in tumors. However, macrophages and cancer‐associated fibroblasts (CAFs) that are present within tumors limit the efficiency of spatiotemporal delivery. To overcome this limitation, glutathion pulse therapy is designed to promote reduction‐sensitive Larotaxel (LTX) prodrug NPs to escape the phagocytosis of macrophages and penetrate through the stromal barrier established by CAFs in the murine triple negative breast cancer model. This therapy improves the penetration of NPs in tumor tissues as well as the accumulation of LTX in cancer cells, and remodels the immunosuppressive microenvironment to synergize PD‐1 blockade therapy. More importantly, a method is established that can directly observe the biodistribution of NPs between different cells in vivo to accurately quantify the target drugs accumulated in these cells, thereby advancing the spatiotemporal delivery research of NPs at the “cellular level.”

## Graphical abstract

Schematic diagram of glutathione (GSH) pulse therapy is shown. GSH pre‐injections remodel the tumor stromal and immunosuppressive microenvironment, which enhance the penetration of nanoparticles (NPs) and synergize the PD‐1 inhibitor therapy. Moreover, the GSH injection at the maximum LTX‐SS‐CA NPs accumulation point significantly promotes the cytotoxic drug Larotaxel to be delivered into cancer cells and reflects remarkable antitumor outcomes.

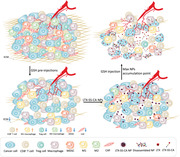

## 1 Introduction

The role of nanomedicine in cancer therapy is growing and several nanosized particles (NPs) have been approved by the FDA.^[^ ^1^, ^2^ ^]^ Many so‐called spatiotemporal NPs delivery systems have been developed and applied to cancer treatment because the therapeutic efficacy depends on the spatial and temporal distribution of agents within the tumor. In terms of the spatial view, NPs are expected to primarily accumulate in tumor tissues and cancer cells. Regarding the temporal view, the maximum drug release in cancer cells is also expected when NPs accumulate in tumor tissues. Therefore, many strategies, such as antibodies modification^[^ ^3^ ^]^ and sensitive chemical linkers,^[^ ^4^, ^5^ ^]^ have been applied to improve the efficiency of the spatiotemporal delivery of NPs. However, due to the complexity of the tumor microenvironment (TME),^[^ ^6^, ^7^ ^]^ ideal outcomes are difficult to achieve.

Macrophages involved in the TME have reportedly been an important factor that affects the spatiotemporal delivery of NPs, and can engulf the majority of NPs existing in tumor tissues, including poly(lactic‐*co*‐glycolic acid) NPs (PLGA‐NPs) and liposomes with or without modification by polyethylene glycol (PEG).^[^ ^8^, ^9^ ^]^ This phenomenon was observed in the reduction‐sensitive nanoassemblies that were designed and reported in our previous studies.^[^ ^10^, ^11^, ^12^ ^]^ Phagocytosis of macrophages within the TME may nullify the effects of NPs in spite of them having been delivered into the tumor tissues, thereby significantly limiting the release of cytotoxic drugs into cancer cells.^[^ ^8^ ^]^ In addition, cancer‐associated fibroblasts (CAFs) within the TME are a key factor that limit the spatiotemporal delivery of NPs.^[^ ^13^ ^]^ CAFs are activated by transforming growth factor‐*β* (TGF‐*β*) that is secreted from cancer cells and can produce dense extracellular matrix (ECM) to build a physical stromal barrier that restricts the penetration of NPs.^[^ ^14^, ^15^ ^]^ Based on these studies, the research level for the spatiotemporal delivery of NPs merely pausing on the “tumor tissues level” is not sufficient, and should be advanced to the “cellular level” to focus on delivering more cytotoxic drug into cancer cells.

According to our previous study,^[^ ^11^ ^]^ sulfur atoms located in the *α*‐position of the ester bond of disulfide‐bond‐linked prodrug nanoassemblies showed better antitumor activities than the *β*‐position NPs and *γ*‐position NPs. This phenomenon mainly due to the *α*‐position disulfide bond being easier to disassemble into small molecules and release cytotoxic drugs. We thought about whether the glutathione (GSH) injection at the point of maximum accumulation of reduction‐sensitive NPs could promote these reduction‐sensitive NPs disassemble into small molecules, which might escape from the engulfing by macrophages through simple diffusion. In addition, this combination also may induce maximum release of the cytotoxic drug into cancer cells. Moreover, based on the oxidative stress state within CAFs, they could secret immunosuppressive cytokines, such as TGF‐*β*, interleutin‐6 (IL‐6), IL‐10, IL‐15, C‐C motif chemokine 2 (CCL2), and CCL17, to help cancer cells establish an immunosuppressive microenvironment to escape the attack from the immune system and immunotherapies, including PD‐1/PD‐L1 checkpoint inhibitors.^[^ ^15^ ^]^ GSH injections are clinically utilized to treat liver fibrosis, which mainly depends on the regulation of the redox state within liver fibroblasts.^[^ ^16^, ^17^ ^]^ Therefore, GSH injections may have a positive effect on the tumor‐stromal and immunosuppressive microenvironment induced by CAFs, which will benefit PD‐1/PD‐L1 checkpoint inhibitor therapy.^[^ ^18^ ^]^

To test our hypotheses, three types of unreported reduction‐sensitive Larotaxel (LTX) prodrug NPs with *α*, *β*, and *γ* disulfide bonds were synthesized and fabricated. Subsequently, the therapeutic outcomes of GSH pre‐injections and injections at the maximum accumulation point of these NPs, named GSH pulse therapy, were investigated in a murine triple‐negative breast cancer model. More importantly, a method that could directly observe the changes of cumulative drugs among different cells was established and the amount of these drugs within target cells was quantified using flow cytometry and cell sorting (FACS), matrix‐assisted laser desorption/ionization mass spectrometry imaging (MALDI‐MSI), and confocal laser scanning microscopy technologies, which advanced spatiotemporal delivery of NPs at the “cellular level.”

## 2 Results

### 2.1 Preparation and Characterizations of LTX‐SS‐CA Prodrug NPs

**Figure 1:**
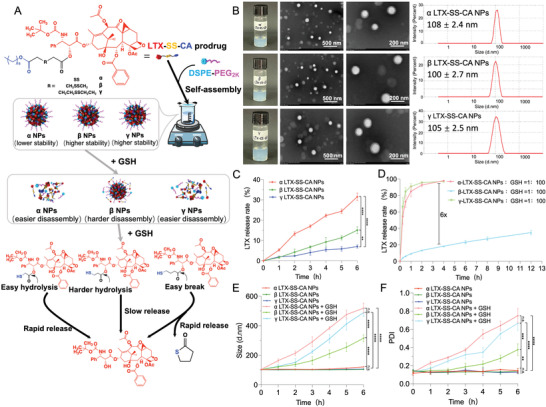
Characterization of reduction‐sensitive LTX‐SS‐CA prodrug nanoparticles. A) Schematic representation of the one‐step nanoprecipitation method and the mechanism of reduction‐responsiveness of these nanoassemblies. B) Photos, hydrodynamic sizes, and transmission electron microscope (TEM) images of LTX‐SS‐CA nanoparticles (NPs). C) Drug release of LTX‐SS‐CA NPs after incubation with rat plasma at 37 °C (*n* = 3). D) In vitro drug release of LTX‐SS‐CA NPs in the presence of various concentrations of glutathione (GSH) (*n* = 3). E,F) Colloidal stability of NPs after incubation with PBS (10% rat plasma V V^−1^) with or without GSH (1:100) at 37 °C (*n* = 3). Statistical significance: n.s. = not significant, *P<0.05, ** *P*<0.01, *** *P*<0.001, and **** *P*<0.0001.

The synthetic routes of three disulfide bond‐bridged LTX and cetyl alcohol (CA) prodrugs are shown in Figure S1 (Supporting Information), and are named *α* LTX‐SS‐CA, *β* LTX‐SS‐CA, and *γ* LTX‐SS‐CA based on the different positions of disulfide‐bonds on the ester bonds. The structures of these three new prodrugs were confirmed by LC‐MS and ^1^H NMR (Figures S2–S4, Supporting Information). Next, hydrophobic LTX‐SS‐CA prodrugs were fabricated to uniform NPs in deionized water using a one‐step nanoprecipitation method (**Figure** **1**A). Furthermore, DSPE‐PEG_2k_ [30% (w/w)] was utilized to enhance the colloidal stability. As shown in Figure 1B and Table S1 (Supporting Information), three types of LTX‐SS‐CA prodrug NPs (*α* NPs, *β* NPs, and *γ* NPs) had spherical structures with an average particle‐size of ≈100 nm. In addition, because the nanocarriers and cargos both were prodrugs, the drug‐loading efficiency [59.5% for *α* NPs, 57.8% for *β* NPs, and 56.4% *γ* NPs (wt%)] was significantly higher than commonly encapsulated nanoformulations (usually less than 10%) (Table S1, Supporting Information).

Next, the plasma stability and reduction‐responsiveness of these three prodrug NPs were investigated. As shown in Figure 1C, the drug‐release rate of *α* NPs was faster than that of *β* NPs and *γ* NPs in rat plasma, which means that *α* NPs have a relatively lower stability.^[^ ^11^ ^]^ And based on Figure 1D and Figure S5 (Supporting Information), the molar ratio of NPs and GSH at a ratio of 1:100 induced the drastic release of LTX. The structure of released LTX was confirmed by ^1^H NMR (Figure S6, Supporting Information). In addition, *α* NPs and *γ* NPs released ≈70% of LTX within NPs in 30 min and 90% in 4 h, which was almost six times higher than that of *β* NPs (Figure 1D). These findings reflected the good reduction‐responsiveness within *α* NPs and *γ* NPs. The results showed that the ester bond within the *α* LTX‐SS‐CA prodrug was the closest to hydrophilic thiol, which induced the fastest release of LTX. For *γ* NPs, its high reduction‐responsiveness mainly contributed to the generated thiol in *γ* disulfide bonds that formed a five‐member ring thioacetone through intramolecular nucleophilic acyl substitution on the ester moiety, which facilitated the release of LTX (Figure 1A). Moreover, the colloidal stability of these prodrug NPs (0.1 × 10^−3^ m) in the presence of 10 × 10^−3^ m GSH was also examined. Shown in Figure 1E,F is the average diameter and PDI of these three NPs, which were both increased in the presence of GSH. However, *α* NPs and *γ* NPs disassembled more rapidly than *β* LTX‐SS‐CA NPs in the presence of GSH.

We examined the reduction‐responsiveness of three types of LTX‐SS‐CA NPs and the results indicated that these NPs released LTX and disassembled easier in the presence of GSH, especially for *α* NPs and *γ* NPs, which showed the most sensitive reduction‐responsiveness.

### 2.2 Cytotoxicity and Cellular Uptake

**Figure 2:**
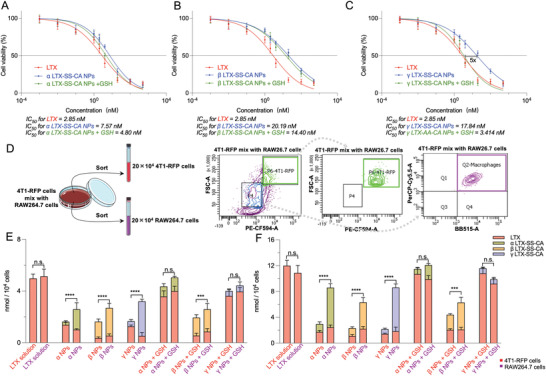
Cytotoxicity and cellular uptake of LTX‐SS‐CA nanoparticles. Cytotoxicity against 4T1 cells by A) *α* LTX‐SS‐CA NPs, B) *β* LTX‐SS‐CA NPs, and C) *γ* LTX‐SS‐CA nanoparticles (NPs) alone and with glutathione (GSH) after 48 h. 37 °C (*n* = 3). D) 4T1‐RFP cells were mixed with RAW264.7 cells and incubated with Larotaxel (LTX) solution and prodrug NPs, then cells were sorted from the P6 gate and Q2 gate, correspondingly. The cellular uptake of the sorted 4T1‐RFP cells and RAW264.7 cells that were incubated for E) 2 h and F) 8 h were measured (*n* = 3). Statistical significance: n.s. = not significant, *P<0.05, ** *P*<0.01, *** *P*<0.001, and **** *P*<0.0001.

The cytotoxicity of prodrug NPs on 4T1 cells was estimated through the MTT method. The IC_50_ values are shown at the bottom of **Figure** **2**A–C. The IC_50_ of the prodrug NPs was reduced by adding GSH (mol‐ratio of prodrugs and GSH was 1:100), especially for *γ* LTX‐SS‐CA NPs, for which the IC_50_ was reduced almost five times in the presence of GSH compared to using *γ* LTX‐SS‐CA NPs alone. The results indicated the faster drug release induced by GSH, which was consistent with the drug release results as shown in Figure 1D.

Regarding the cellular uptake of NPs, to investigate its variations with or without GSH between cancer cells and macrophages, 4T1‐RFP cells and RAW264.7 cells were selected as model cells and the amount of LTX and prodrugs accumulated in RAW264.7 cells mixed with 4T1‐RFP cells were measured at a quantity ratio of 1:1. In addition, a novel FACS method was established to sort target cells (Figures S7–S10, Supporting Information). This method was mainly based on the characteristics of RAW264.7 cells and 4T1‐RFP cells. For RAW264.7 cells, accurate sorting of these cells was mainly based on three types of specific antigens located on the cells, including CD45, CD11b, and F480, which are specific antigens for macrophages.^[^ ^19^ ^]^ For 4T1‐RFP cells, due to the red fluorescent proteins (RFP) within the cells, that can be excited at 488 or 532 nm laser and are detected around 588 nm, the PE‐CF594 channel can be utilized to sort these cells from cells without RFP, thus the P6 gate was set in PE‐CF594 to sort 4T1‐RFP cells and Q2 gates were set to sort RAW264.7 cells (Figure 2D). As shown in Figure S7 (Supporting Information), RAW 264.7 cells were successfully sorted from the Q2 gate, and no signal was observed in the P6 gate. In addition, as shown in Figure S8 (Supporting Information), 4T1‐RFP cells were sorted from the P6 gate and no signal was observed in the Q2 gate and Q2‐2 gate, which indicated that 4T1‐RFP cells did not affect the sorting of RAW264.7 cells. Next, for 4T1‐RFP cells mixed with RAW264.7 cells, as shown in Figure S9 (Supporting Information), RAW264.7 cells were successfully sorted through the Q2 gate from the mixed‐up cells’ population, and 4T1‐RFP cells were purified through the P6 gate. Although several signals are present in the Q2‐2 gate, the total purity of 4T1‐RFP cells was over 99%.

Therefore, based on this FACS method, the amount of LTX and prodrugs in these cells were measured through LC‐MS/MS and the changes in cellular uptake were investigated among various treatments. As shown in Figure 2E,F, the accumulation of LTX and prodrugs in RAW264.7 cells was significantly higher than those in 4T1‐RFP cells when incubated with NPs alone after 2 and 8 h. However, when the cells were incubated with NPs and GSH (mol‐ratio of prodrugs and GSH was 1:100), the uptake of LTX and prodrugs of 4T1‐RFP cells was markedly increased for *α* NPs and *γ* NPs. However, for *β* NPs, the cellular uptake of RAW264.7 cells was markedly higher than that of 4T1‐RFP cells. These results mainly contributed to the difference in reduction‐responsiveness among these prodrug NPs. Due to the high reduction responsiveness for *α* NPs and *γ* NPs, these NPs could dramatically disassemble into small molecules under the action of GSH and permeate into cells through a free diffusion effect like the LTX solution preparation. However, *β* NPs cannot efficiently disassemble into small molecules despite adding the GSH, thus its cellular uptake behavior was similar to the addition of *β* NPs alone.

Taken together, we found that adding excessive GSH can enhance the cytotoxicity of the three reduction‐sensitive NPs, especially for *γ* NPs, and promote the disassembly of *α* LTX‐SS‐CA NPs and *γ* LTX‐SS‐CA NPs, which enhanced the cellular uptake of LTX and prodrugs in 4T1‐RFP cells.

### 2.3 In Vivo Antitumor Efficacy of Prodrug NPs

**Figure 3:**
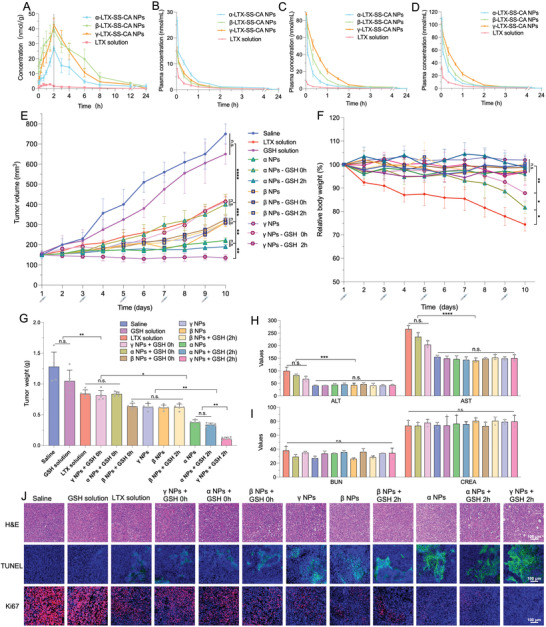
Pharmacokinetic profiles and in vivo antitumor efficacy of Larotaxel and LTX‐SS‐CA nanoparticles. A) Tumor concentration–time profiles of Larotaxel (LTX), *α*, *β*, and *γ* prodrugs in Balb/c mice after intravenous injection of LTX solution or LTX‐SS‐CA prodrug nanoparticles (NPs) (7.2 µmol kg^−1^) (*n* = 5). Plasma concentration–time profiles of B) the prodrugs, C) the released LTX, and D) their sum. E) Tumor growth profiles and G) tumor weights after various treatments (7.2 µmol kg^−1^) (*n* = 5). F) Changes in mouse body weight after various treatments (*n* = 5). H) Hepatic and I) renal function parameters of mice after administrations (*n* = 5). (G)–(I) share the same color coding. ALT, alanine aminotransferase (U L^−1^); AST, aspartate aminotransferase (U L^−1^); BUN, blood urea nitrogen (mmol L^−1^); CREA, creatinine (µmol L^−1^). J) H&E, TUNEL, and Ki67 staining of tumors from all groups. Statistical significance: n.s. = not significant, *P<0.05, ** *P*<0.01, *** *P*<0.001, and **** *P*<0.0001.

Before investigating the antitumor effect of this “GSH pulse therapy”, tumor pharmacokinetics of these prodrug NPs in 4T1 tumor‐bearing mice were studied to confirm “the time of maximum accumulation of NPs” in tumors, and GSH would be administrated at that time. As shown in **Figure** **3**A, at 2 h after addition, NPs accumulation reached a maximum. It was also found that *α* NPs (Figure S11A, Supporting Information) released more LTX in tumors compared to *β* NPs (Figure S11B, Supporting Information) and *γ* NPs (Figure S11C, Supporting Information), and the accumulation of *β* LTX‐SS‐CA prodrug and *γ* LTX‐SS‐CA prodrug was higher than that of *α* LTX‐SS‐CA prodrug, thereby indicating that the stability of *β* and *γ* NPs was higher than that of *α* NPs. The plasma pharmacokinetic studies showed similar results (Figure 3B–D; Tables S2 and S3, Supporting Information).

Next, dosing regimens were designed for 4T1 tumor‐bearing mice: 1) the NPs were given by three types of administration: NPs injection alone (NPs), NPs and GSH injection at the same time (NPs + GSH 0 h), and injecting NPs first, followed by the GSH injection after 2 h [NPs + GSH 2 h] (GSH pulse therapy); 2) administration of three types of solutions, namely, saline, LTX, and GSH.

The in vivo therapeutic outcomes of these dosing regimens are presented in Figure 3E–G. The saline solution and GSH solution‐treated groups had the fastest tumor growth and there were no obvious differences between these two groups. In addition, the antitumor activities were found to be similar for the LTX solution group, (*α* NPs + GSH 0 h) group, and (*γ* NPs + GSH 0 h) group, which indicated that *α* NPs and *γ* NPs rapidly released LTX with the function of GSH in circulation. Then, because *β* NPs could not efficiently release LTX under the action of GSH, the (*β* NPs + GSH 0 h) group and (*β* NPs + GSH 2 h) group had an analogous antitumor activity to that of the *β* NPs group. The *α* NPs group and (*α* NPs + GSH 2 h) group had better antitumor activities than *β* NPs and *γ* NPs. Notably, the (*γ* NPs + GSH 2 h) group had the best antitumor activity among all the groups. This outcome mainly contributed to the GSH pulse therapy of *γ* NPs released the highest dosage of LTX into cancer cells. As for the changes in relative body weight (Figure 3F), notable body weight loss was observed in the LTX solution group, (*α* NPs + GSH 0 h) group and (*γ* NPs + GSH 0 h) group, while no markedly difference was observed among the other groups. The premature release of LTX within the three groups might be the main reason resulting in the loss of weight. The side effects were also reflected in the results of hepatic functions and hematoxylin and eosin (H&E) staining of liver sections (Figure 3H and Figure S12, Supporting Information). In addition, no obvious changes in renal function were observed in these groups (Figure 3I). Furthermore, H&E, TUNEL, and Ki67 staining of tumor tissue sections also showed that the (*γ* NPs + GSH 2 h) group had the most widespread apoptosis of cancer cells (Figure 3J).

### 2.4 MALDI‐MSI and Confocal Microscope Imaging for Tumor tissues

To investigate the underlying mechanism behind the antitumor activities of these administrations, MALDI‐MSI was employed to observe the distribution of LTX and the prodrugs within tumor tissues and confocal microscopy was performed to obtain the distribution of cancer cells (4T1‐RFP cells) and macrophages within tumor tissues at 2.5 h after the administration, which was 0.5 h after the GSH administration in the GSH pulse groups.

**Figure 4:**
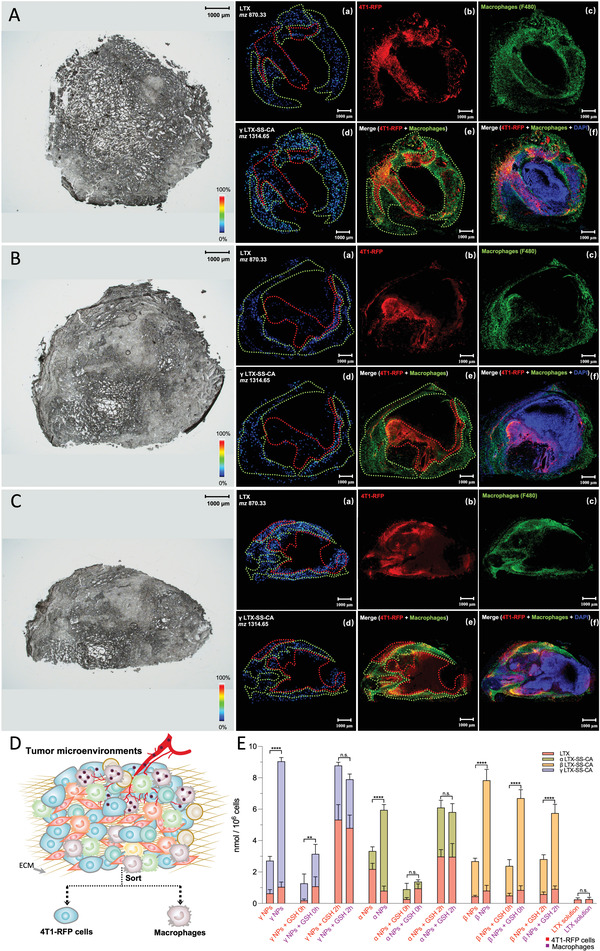
MALDI‐MSI, fluorescence images, and FACS for tumors. Optical images, PFOA‐related MALDI signal ([M + K^+^] at m/z 870.33 for Larotaxel (LTX); [M + K^+^] at m/z 1314.65 for *γ* LTX‐SS‐CA prodrug) and fluorescence images (RFP, red; F480, green; DAPI, blue) (macrophages, green outline; 4T1‐RFP cells, red outline) of tumor tissues from tumor‐bearing mice after treatment with A) *γ* nanoparticles (NPs), B) (*γ* NPs + GSH 0 h), and C) (*γ* NPs + GSH 2 h). D) The 4T1‐RFP cells and macrophages within these tumors were sorted, and E) the amount of LTX and prodrugs in these cells was measured. Statistical significance: n.s. = not significant, *P<0.05, ** *P*<0.01, *** *P*<0.001, and **** *P*<0.0001.

As shown in **Figure** **4**A (*γ* NPs), S13A (*α* NPs), and S14A (*β* NPs), when NPs were administrated alone, most of the NPs were preferentially accumulated in macrophages, and only a few were distributed into 4T1‐RFP cells. In addition, the signal of LTX from *α* NPs (Figure S13Aa, Supporting Information) was higher than that of *γ* NPs (Figure 4Aa) and *β* NPs (Figure S14Aa, Supporting Information), which could explain why *α* NPs have better antitumor activities than *β* NPs and *γ* NPs. As for the administration of (NPs + GSH 0 h), signals of LTX and prodrugs of (*α* NPs + GSH 0 h) group and (*γ* NPs + GSH 0 h) group were found to be markedly lower than signals after injections of *α* NPs and *γ* NPs alone and similar to the administration of LTX solution (Figure S15, Supporting Information), which could explain the similar therapeutic outcomes of these three groups. However, the signals of (*β* NPs + GSH 0 h) (Figure S14B, Supporting Information) were stronger than those of the (*α* NPs + GSH 0 h) group and (*γ* NPs + GSH 0 h) group, which mainly contributed to the more difficult disassembly of *β* NPs. Moreover, the lower reductive responsiveness may be one of the reasons that the (*β* NPs + GSH 0 h) group had a better antitumor activity than the (*α* NPs + GSH 0 h) group and (*γ* NPs + GSH 0 h) group. In addition, for the GSH pulse groups, we were surprised to find that the LTX signal of the (*γ* NPs + GSH 2 h) group (Figure 4Ca) and (*α* NPs + GSH 2 h) group (Figure S13Ca, Supporting Information) was significantly higher than that of two NPs injections alone (Figure 4Aa and Figure S13Aa, Supporting Information). Furthermore, no marked differences in distribution were observed between macrophages and 4T1‐RFP cells, which indicated that the GSH pulse therapy increased the amount of LTX and prodrugs accumulated in 4T1‐RFP cells compared to that of NPs injections alone. This was an important observation for the notable antitumor activities of these two groups. In addition, based on the higher stability of *γ* NPs, *γ* NPs can accumulate more than *α* NPs, leading to the highest LTX signal and the best therapeutic outcome among all groups. However, for *β* NPs, due to the comparatively lower reduction responsiveness, and despite adding GSH at the point of maximum accumulation, LTX was hardly released.

To further investigate the changes in the amount of LTX and prodrugs between 4T1‐RFP cells and macrophages, the FACS method (Figure S10, Supporting Information) as illustrated above was utilized to sort 4T1‐RFP cells and macrophages from tumor tissues and the amount of LTX and prodrugs within these cells was measured through LC‐MS/MS (Figure 4D). As shown in Figure 4E, the results were consistent with MADLI‐MSI and confocal microscopy images. The accumulation of LTX and prodrugs in macrophages was significantly higher compared to those within 4T1‐RFP cells in *α* NPs and *γ* NPs injections alone groups and all *β* NPs groups. In addition, the amount of LTX and prodrugs was markedly increased in 4T1‐RFP cells of (*α* NPs + GSH 2 h) and (*γ* NPs + GSH 2 h) groups, and among the groups, the (*γ* NPs + GSH 2 h) group showed the largest accumulation of LTX and prodrug in 4T1‐RFP cells.

Therefore, the above‐mentioned results demonstrated that GSH pulse therapy significantly improved the spatiotemporal delivery of reduction‐sensitive NPs, especially for *γ* LTX‐SS‐CA NPs, which efficiently increased the amount of LTX and prodrugs within 4T1‐RFP cells.

### 2.5 Effect of GSH on Tumor Stromal Microenvironment

**Figure 5:**
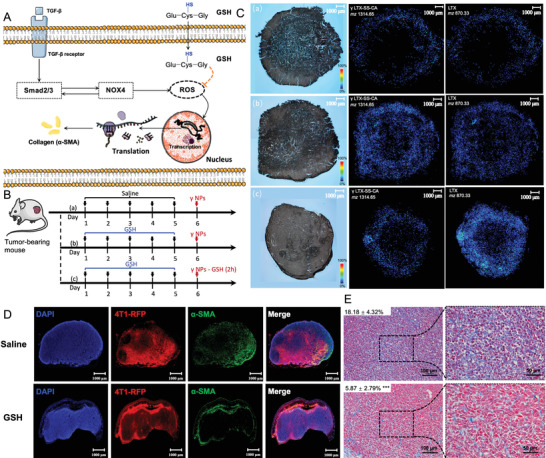
Effects of glutathione on cancer‐associated fibroblasts and the delivery of NPs. A) Schematic illustration of the mechanism of glutathione (GSH) inhibits the production of collagen (*α*‐SMA) by improving the oxidative stress state of cancer‐associated fibroblasts (CAFs). B) Tumor treatment scheme for saline and GSH following *γ* NPs injection with or without GSH. C) Optical images and PFOA‐related MALDI signal ([M + K^+^] at m/z 870.33 for LTX; [M + K^+^] at m/z 1314.65 for *γ* LTX‐SS‐CA prodrug) of tumor tissues from tumor‐bearing mice that were treated according to the treatment scheme. D) Fluorescence images of tumor tissues (RFP, red; *α*‐SMA, green; DAPI, blue). E) Masson's trichrome staining and quantification of collagen deposition are expressed as the percentage of total cell number (*n* = 5). Statistical significance: n.s. = not significant, *P<0.05, ** *P*<0.01, *** *P*<0.001, and **** *P*<0.0001.

In this study, we investigated the characteristics of the distribution of NPs between cancer cells and macrophages and found that GSH pulse therapy of LTX‐SS‐CA NPs could improve the amount of LTX and prodrugs in cancer cells to enhance the antitumor activates of NPs. In addition, we discovered that the distribution of LTX and prodrugs was around the edge of tumor tissues (Figure 4A–C; Figures S13 and S14, Supporting Information), thereby indicating the penetration depth of NPs was limited. It has been reported that CAFs may be the critical factor in this phenomenon.^[^ ^13^, ^20^ ^]^ CAFs activated by TGF‐*β* secreted from cancer cells could produce a dense ECM to increase the interstitial fluid pressure within the tumor and limit the delivery of NPs.^[^ ^21^ ^]^ This activated status of CAFs was mainly achieved through reactive oxygen species (ROS) generated by the TGF‐*β* signaling pathway,^[^ ^22^ ^]^ which is illustrated in **Figure** **5**A. GSH, an important reducer in vivo, was found to have a positive effect on the delivery of NPs by inhibiting the activation of CAFs.

The inhibitory effect of GSH on ROS production was examined in NIH3T3 cells activated by TGF‐*β* (10 ng mL^−1^). As shown in Figure S16 (Supporting Information), GSH inhibited the ROS production of NIH3T3 cells in a concentration‐dependent manner. We next designed a treatment scheme of saline, GSH solution, and *γ* NPs to observe whether GSH pre‐injections could have a positive effect on the penetration of NPs (Figure 5B). After five consecutive daily injections of saline and GSH solution, mice were sacrificed and tumors were harvested for further investigation. The MADLI‐MSI results (Figure 5C) showed that the penetration of NPs in two GSH pre‐injection groups (Figure 5Cb,c) was significantly deeper than that of the saline pre‐injection group (control) (Figure 5Ca), and reached the “core” of the tumor.

Next, we further investigated the underlying mechanism behind the improvement of GSH solution on the penetration of NPs. As shown in Figure 5D and Figure S17 (Supporting Information), immunofluorescence staining showed that the expression of *α*‐SMA, the main component of collagen, in the GSH group was markedly reduced. Furthermore, collagen was also the main component of the ECM. To visualize the collagen deposition within tumor tissues, Masson's trichrome staining was performed (Figure 5E) and the results showed that the collagen density of the GSH pre‐injections group was significantly decreased than saline group. In addition, because the NADPH oxidase (NOX) enzyme and Smad2/3 protein are key proteins within the pathway of the production of *α*‐SMA induced by TGF‐*β*,^[^ ^23^, ^24^ ^]^ Western blot (WB) analysis (Figure S18, Supporting Information) was used to analyze their expressions levels. The data showed that the expression of NOX4, *α*‐SMA, P‐Smad2, and P‐Smad3 were markedly downregulated in the GSH group than saline group, which confirmed that GSH pre‐injections could inhibit collagen production by regulating oxidative stress within CAFs.

### 2.6 Effect of GSH on Tumor Immunosuppressive Microenvironment

**Figure 6:**
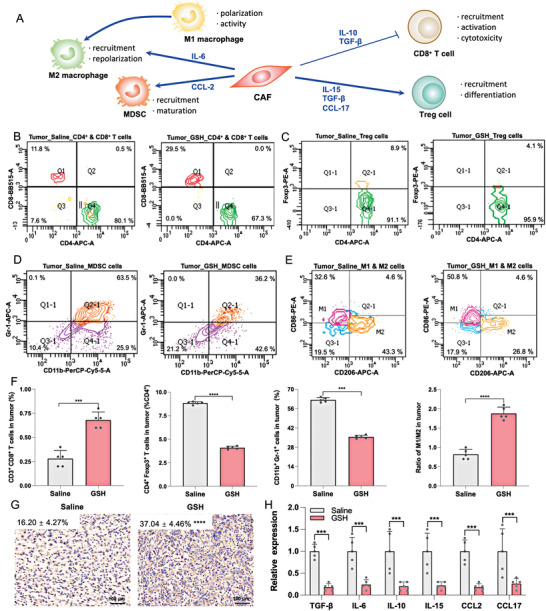
The changes induced by glutathione on the tumor immune microenvironment. A) Schematic illustration of the immunosuppressive microenvironment established by cancer‐associated fibroblasts (CAFs). B) Analysis of CD4^+^ and CD8^+^ cells in the tumor after being treated with saline and glutathione (GSH) by flow cytometry (*n* = 5). Analysis of C) regulatory T cells (Tregs), D) myeloid‐derived suppressor cells (MDSCs), and E) M1 & M2 cells in the tumor after being treated with saline and GSH by flow cytometry (*n* = 5). F) Quantification of the results of flow cytometry (*n* = 5). G) Comparison of PD‐1 expression of tumors after saline and GSH treatments through immunocytochemical staining (*n* = 5). H) Changes of immunosuppressive cytokines levels after saline and GSH treatment (*n* = 3). Statistical significance: n.s. = not significant, *P<0.05, ** *P*<0.01, *** *P*<0.001, and **** *P*<0.0001.

We further investigated whether GSH could remodel the tumor immunosuppressive microenvironment induced by CAFs within 4T1 breast tumors. As shown in **Figure** **6**A, activated CAFs could inhibit the proliferation and activity of CD8^+^ T cells and increased the number of immunosuppressive cells, including regulatory T cells (Tregs), myeloid‐derived suppressor cells (MDSCs), and M2 macrophages, through the secretion of immunosuppressive cytokines, including TGF‐*β*, IL‐6, IL‐10, IL‐15, CCL2, and CCL17. These immunosuppressive cells and cytokines commonly established the tumor immunosuppressive microenvironment.

T cells, especially CD8^+^ T cells, are the main antitumor cells within the immune system, but the physical barrier and immunosuppressive microenvironments induced by CAFs limit the infiltration of T cells.^[^ ^18^, ^20^ ^]^ As shown in previous studies,^[^ ^25^, ^26^ ^]^ GSH can enhance the number and activity of CD8^+^ T cells. As a result, the infiltration of CD8^+^ T cells within the tumor was significantly improved in the GSH group (Figure 6B; Figure S19, Supporting Information; and Figure 6F). Moreover, the number of recruited Tregs (Figure 6C; Figure S19, Supporting Information; and Figure 6F) and MDSCs (Figure 6D; Figure S20, Supporting Information; and Figure 6F) was markedly reduced in the GSH group. Pre‐injections of GSH also promoted the M2 macrophage phenotype to switch to the pro‐inflammatory M1 macrophage phenotype (Figure 6E,F, and Figure S21, Supporting Information). Next, immunocytochemical staining of tumor tissues was performed to measure the expression level of PD‐1 (Figure 6G). The results showed that GSH pre‐injections upregulated the expression of PD‐1, which might be due to the up‐regulation of CD8^+^ T cells and improvement of the immunosuppressive microenvironment. The conditions of up‐regulation of CD8^+^ T cells and PD‐1 expression within tumor microenvironments were also suitable for the application of PD‐1 blockade therapy. Moreover, the level of immunosuppressive cytokines in the GSH group was downregulated compared to the saline group, thereby suggesting that reversal of the immunosuppressive microenvironment may contributed to the inhibition of CAFs induced by GSH pre‐injections (Figure 6H).

### 2.7 In Vivo Antitumor Efficacy of GSH Pulse Therapy Combined with PD‐1 Blockade Therapy

**Figure 7:**
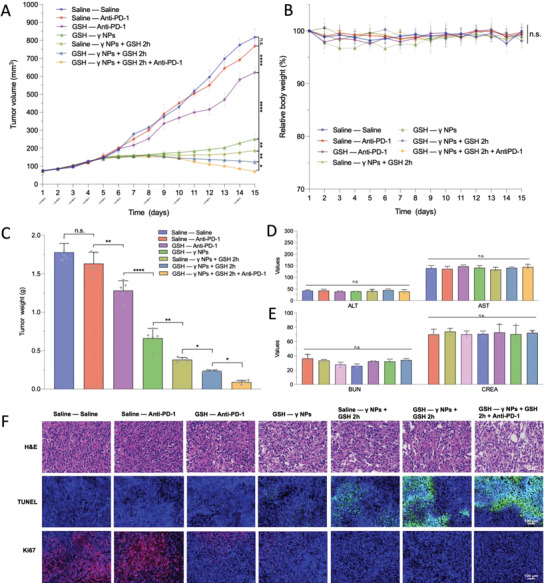
Combination of *γ* NPs’ glutathione pulse therapy and PD‐1 blockade therapy in the 4T1 tumor model. A) Tumor volume, B) changes in body weight, and C) tumor weight of different groups (*n* = 5). D) Hepatic and E) renal function parameters of different groups (*n* = 5). (C)–(E) share the same color coding. F) H&E, TUNEL, and Ki67 staining of tumors from different groups. Statistical significance: n.s. = not significant, *P<0.05, ** *P*<0.01, *** *P*<0.001, and **** *P*<0.0001.

Based on the effects of GSH pre‐injections on the tumor stromal microenvironment and immunosuppressive microenvironment, we further investigated the antitumor effect of GSH pulse therapy of *γ* NPs combined with PD‐1 blockade therapy (2 mg kg^−1^). As shown in **Figure** **7**A–C, no differences were observed between the anti‐PD‐1 monotherapy group and the control group due to the immunosuppressive microenvironment within the tumor, which had the fastest tumor growth. As for the anti‐PD‐1 combined with GSH pre‐injections group, there was a significant improvement in the antitumor activity of anti‐PD‐1, which might be contributed to reversal of the immunosuppressive microenvironment by GSH pre‐injections. GSH pre‐injections also enhanced the therapeutic outcomes of the GSH pulse therapy of *γ* NPs. Notably, GSH pre‐injections combined with GSH pulse therapy and anti‐PD‐1 blockade showed the best antitumor activity among all groups, which indicated that improvement of the tumor stromal and immunosuppressive microenvironment significantly enhanced the antitumor activities of *γ* NPs and anti‐PD‐1. Regarding the safety of these administrations, no significant changes were observed in relative body weight and hepatorenal functions among all groups (Figure 7B,D,E). Furthermore, H&E, TUNEL, and Ki67 staining of tumor tissues (Figure 7F) showed that GSH pre‐injections combined with *γ* NPs’ GSH pulse therapy and anti‐PD‐1 blockade induced the highest level of apoptosis within tumor cells compared to other groups.

## 3 Discussion

Spatiotemporal delivery of NPs at the “cellular level,” with the aim to deliver as much cytotoxic drug into cancer cells as possible during the residence time of NPs in tumors, is crucial for the development of cancer nanomedicine. However, the complexity of the TME limits its efficiency, especially for macrophages and CAFs. Li et al. compared the biodistribution of nanoparticulate albumin‐bound paclitaxel (nab‐PTX) and other commercial NPs, including *β*‐Cyclodextrin nanoparticles (CDNPs), PLGA‐NPs, and liposomes.^[^ ^8^ ^]^ They found that nab‐PTX mainly accumulated in cancer cells due to rapid disassembly and the targeting function of albumin, while the other NPs preferentially accumulated in macrophages. Moreover, Xu et al. designed nano‐puerarin to deactivate CAFs by inhibiting the ROS production, which is the key downstream mediator of the TGF‐*β* profibrogenic pathway.^[^ ^20^ ^]^ In the current study, we demonstrated that the higher the stability of NPs (*β* NPs and *γ* NPs), the more they would be engulfed by macrophages within tumor tissues. Lei et al. showed that the presence of disulfide bonds might reduce the stability of chemical structures, which would enhance drug release in the circulation, therefore, the position of disulfide within the prodrugs bond should be carefully designed.^[^ ^27^ ^]^ Their finding was consistent with the results of *α* NPs in the current study. Regarding *α* NPs with a relatively lower stability, it was found that they had a better antitumor activity than *β* NPs and *γ* NPs due to the rapid disassembly into small molecules. However, the accumulation of *α* NPs within tumors was not as high as that of *β* NPs and *γ* NPs. Therefore, we considered a strategy that could maintain the high accumulation of NPs and release more cytotoxic drugs at the same time.

Considering the interventions of macrophages and CAFs for the spatiotemporal delivery of NPs and being inspired by the disassembly of nab‐PTX and *α* NPs and oxidative stress within CAFs, we designed the GSH pulse therapy to promote the disassembly of reduction‐sensitive NPs and improve oxidative stress within CAFs. This therapy consists of five consecutive days of GSH pre‐injection, five injections of reduction‐sensitive NPs followed by GSH injections at the time of maximum accumulation of reduction‐sensitive NPs in next every other day. As illustrated by the results above, GSH pre‐injections significantly promoted the penetration of *γ* NPs into the core of tumors, while *γ* NPs of the saline pre‐injections group were merely distributed around the edge of the tumors. This outcome mainly contributed to the inhibition of GSH on CAFs, which could reduce the production of collagen within the ECM. A GSH injection at the time of maximum accumulation of *γ* NPs could markedly increase the accumulation of LTX and *γ* prodrugs in cancer cells, which enhanced the antitumor activity of *γ* NPs by promoting the *γ* NPs to rapidly disassemble into small molecules to escape the engulfing of macrophages and increase the amount of LTX and prodrugs in cancer cells. The strategy may also be extended to other reduction‐sensitive drug delivery systems.

In this study, the immune regulation of GSH was also emphasized, and the tumor immunosuppressive microenvironment was remodeled to synergize the PD‐1 blockade therapy. In fact, the regulation of GSH on T cell proliferation was found as early as 1990 and GSH injections have been widely utilized in cancer patients to improve their immune systems and reduce the side effects of chemotherapy.^[^ ^25^ ^]^ The redox regulation of immunometabolism has also been emphasized in recent studies.^[^ ^28^, ^29^ ^]^ To our knowledge, the use of GSH to improve the spatiotemporal delivery of reduction‐sensitive NPs has not been reported in previous studies.

More importantly, we creatively presented the concept of “spatiotemporal delivery of NPs at the cellular level.” Most previous studies that focused on the spatiotemporal delivery of NPs utilized the amount of NPs or drugs accumulated in tumor tissues or the tissues distribution to estimate the therapeutic outcomes of NPs. However, with more thorough research on the TME, we gradually realized that a higher accumulation of NPs within a tumor might not be equivalent to better antitumor activities. These NPs would be engulfed by macrophages in tumors or just distributed among the edge of tumors and could not penetrate into the core of tumors. The cytotoxic drugs involved in NPs could not efficiently be released into cancer cells. Therefore, the spatiotemporal delivery of NPs paused at the “tissue level” and did not meet the needs of further advanced nanomedicine. Effective methods to support research on the spatiotemporal delivery of NPs at the cellular level are warranted. Therefore, we established a method that not only can observe and quantify the biodistribution of drugs among different cells in tumors but can also investigate the penetration of NPs in tumor tissues through MALDI‐MSI, FACS, and confocal microscope technologies, which advances research on the spatiotemporal delivery of NPs at the “cellular level.” MALDI‐MSI is a novel imaging technology that can map the biodistribution of various molecules within a tissue, which is ideal for research on the drug delivery system.^[^ ^30^ ^]^ We combined this method with confocal microscope and successfully observed changes in the distribution of LTX and LTX‐SS‐CA prodrugs between cancer cells and macrophages. This method can also be expanded to determine the biodistribution of any small molecule among target cells. In addition, the combination of FACS with LC‐MS/MS helped us accurately quantify the amount of drugs accumulated in both cancer cells and macrophages. Furthermore, sophisticated transfection technology using fluorescent proteins makes that almost every type of cell can be sorted by FACS. Therefore, the method we established is highly suitable for research on cellular‐level drug delivery in vivo, which not only includes NPs, but also antibody–drug conjugates (ADCs) and other targeted biomaterials.

## 4 Conclusions

In this study, we developed GSH pulse therapy for reduction‐sensitive NPs to promote the spatiotemporal delivery of NPs into cancer cells. The strategy can synergize PD1 blockade therapy. Finally, we established an analytical method that can advance the research of NPs delivery at the “cellular level” by integrating MADLI‐MSI, FACS, and confocal microscope technologies.

## 5 Experimental Section

### Materials

LTX was obtained from Beijing Hexie Weiye Biotechnology Co., Ltd. (Beijing, China). Cetyl alcohol, GSH, 1‐ethyl‐3‐(3‐dimethyl aminopropyl) carbodiimide hydrochloride (EDCI), hydroxy benzotriazole (HoBt), 4‐dimethylamino pyridine (DMAP), and MTT were purchased from Aladdin Co., Ltd. (Shanghai, China). DSPE‐PEG_2k_ was obtained from Shanghai Advanced Vehicle Technology Co., Ltd. 4′6‐Diamidino‐2‐phenylindole (DAPI), TUNEL, and Ki67 assay kit were purchased from Beijing Solarbio Science & Technology Co., Ltd. (Beijing, China). The AF700 antibody, anti‐CD45 antibody, anti‐CD3 antibody, anti‐CD4 antibody, anti‐CD8 antibody, anti‐Foxp3 antibody, anti‐CD11b antibody, anti‐Gr‐1 antibody, anti‐F4/80 antibody, anti‐CD206 antibody, anti‐CD86 antibody, anti‐NOX4 antibody, anti‐P‐Smad2 antibody, anti‐Smad3 antibody, anti‐*α*‐SMA antibody, and anti‐GAPDH antibody were purchased from ABclonal Biotechnology Co., Ltd. (Wuhan, China). Anti‐PD‐1 (Anti‐mouse‐CD279) was purchased from Biolegend Co., Ltd. (Beijing, China). All other reagents utilized in this study were of analytical grade.

### Synthesis of CA‐*α*‐SS‐LTX, CA‐*β*‐SS‐LTX, and CA‐*γ*‐SS‐LTX

2,2′‐Dithiocarboxylic acid (2.11 g, 11.56 mmol), 3,3′‐dithiodipropionic acid (2.43 g, 11.56 mmol), and 4,4′‐dithiodibutyric acid (2.73 g, 11.56 mmol) were dissolved in 10 mL acetic anhydride under the protection of N_2_. After stirring for 2 h under 25 °C, the superfluous acetic anhydride was removed, and then 2,2′‐dicarboxylic anhydride, 3,3′‐dithiodipropionic anhydride, and 4,4′‐dithiodibutyric anhydride were obtained. An equimolar quantity of cetyl alcohol (2.80 g, 11.56 mmol) was added to these three kinds of anhydride, respectively. And HoBt (0.78 g, 5.78 mmol), EDCI (1.11 g, 5.78 mmol), and DMAP (0.07 g, 0.58 mmol) were added to catalyze the reactions. After stirring overnight under 25 °C, CA‐*α*‐SS‐COOH, CA‐*β*‐SS‐COOH, and CA‐*γ*‐SS‐COOH were obtained with a yield of about 60%. CA‐*α*‐SS‐COOH (or CA‐*β*‐SS‐COOH, or CA‐*γ*‐SS‐COOH) (2.85 mmol), Larotaxel (2.37 g, 2.85 mmol), EDCI (0.55 g, 2.85 mmol) was added into this mixture and the whole mixture was stirred for 1 h under nitrogen atmosphere at 0 °C. Then, the mixture was further stirred for 48 h at room temperature. The TLC was used to monitor the process of the reaction and the target prodrugs (LTX‐*α*‐SS‐CA, LTX‐*β*‐SS‐CA and LTX‐*γ*‐SS‐CA) were purified by preparative liquid chromatography using acetonitrile as mobile phase with a yield of about 65%. The structures of these target prodrugs were confirmed by SolariX 7.0 T ESI‐MS (Bruker, Germany) and Bruker AV‐400 NMR Spectroscopy (Bruker, Germany). The *m/z* of LTX‐*α*‐SS‐CA: [M + Na]^+^ calcd for C_65_H_89_NNaO_17_S_2_, 1242.545159; found, 1242.546413. ^1^H NMR (400 MHz, DMSO‐*d*_6_) *δ* 8.01 (d, *J* = 7.3 Hz, 2H), 7.83 (d, *J* = 8.9 Hz, 1H), 7.77–7.73 (m, 1H), 7.68 (t, *J* = 7.3 Hz, 2H), 7.39 (d, *J* = 7.2 Hz, 4H), 7.07 (t, *J* = 6.6 Hz, 1H), 6.10 (s, 1H), 5.78 (t, *J* = 7.6 Hz, 1H), 5.42 (d, *J* = 7.5 Hz, 1H), 5.18 (d, *J* = 8.0 Hz, 1H), 5.05 (t, *J* = 8.4 Hz, 1H), 4.72 (s, 1H), 4.61 (s, 1H), 4.06 (q, *J* = 6.8 Hz, 3H), 3.95 (d, *J* = 8.4 Hz, 1H), 3.84 (s, 3H), 3.72 (s, 2H), 2.32 (s, 1H), 2.26 (s, 3H), 2.10 (s, 3H), 1.99 (dd, *J* = 22.1, 14.8 Hz, 2H), 1.87–1.76 (m, 1H), 1.68 (s, 3H), 1.61–1.55 (m, 2H), 1.50 (d, *J* = 4.4 Hz, 2H), 1.36 (s, 9H), 1.23 (s, 30H), 1.10 (s, 3H), 1.01 (s, 3H), 0.85 (s, 1H). The *m/z* of LTX‐*β*‐SS‐CA: [M + Na]^+^ calcd for C_67_H_93_NNaO_17_S_2_, 1270.575149; found, 1270.577713.^1^ H NMR (400 MHz, DMSO‐*d*_6_) *δ* 8.01 (d, *J* = 7.2 Hz, 2H), 7.84 (d, *J* = 9.3 Hz, 1H), 7.74 (t, *J* = 7.4 Hz, 1H), 7.67 (t, *J* = 7.4 Hz, 2H), 7.39 (q, *J* = 7.5, 7.0 Hz, 4H), 7.10 (t, *J* = 6.3 Hz, 1H), 6.10 (s, 1H), 5.80 (t, *J* = 9.1 Hz, 1H), 5.43 (d, *J* = 7.6 Hz, 1H), 5.14 (d, *J* = 7.6 Hz, 1H), 5.11–5.04 (m, 1H), 4.72 (s, 1H), 4.62 (s, 1H), 4.04 (q, *J* = 6.6 Hz, 3H), 3.95 (d, *J* = 8.5 Hz, 1H), 3.84 (d, *J* = 7.3 Hz, 1H), 2.92 (q, *J* = 6.7 Hz, 4H), 2.80 (t, *J* = 6.5 Hz, 2H), 2.69 (t, *J* = 6.8 Hz, 2H), 2.35–2.28 (m, 1H), 2.26 (s, 3H), 2.10 (s, 3H), 2.02 (dd, *J* = 10.0, 4.6 Hz, 1H), 1.96 (d, *J* = 16.0 Hz, 1H), 1.86 (d, *J* = 9.9 Hz, 1H), 1.68 (s, 3H), 1.54 (dt, *J* = 12.5, 6.5 Hz, 5H), 1.36 (s, 9H), 1.23 (s, 28H), 1.10 (s, 3H), 1.02 (s, 3H), 0.85 (s, 1H). The *m/z* of LTX‐*γ*‐SS‐CA: [M + Na]^+^ calcd for C_69_H_97_NNaO_17_S_2_, 1298.608477; found, 1298.609013.^1^ H NMR (400 MHz, DMSO‐*d*_6_) *δ* 8.00 (d, *J* = 7.4 Hz, 2H), 7.84 (d, *J* = 9.4 Hz, 1H), 7.75 (t, *J* = 7.5 Hz, 1H), 7.68 (t, *J* = 7.4 Hz, 2H), 7.38 (dt, *J* = 12.4, 7.4 Hz, 4H), 7.08 (t, *J* = 7.2 Hz, 1H), 6.10 (s, 1H), 5.79 (t, *J* = 9.3 Hz, 1H), 5.44–5.38 (m, 1H), 5.13 (d, *J* = 8.0 Hz, 1H), 5.05 (t, *J* = 8.9 Hz, 1H), 4.72 (d, *J* = 3.0 Hz, 1H), 4.62 (s, 1H), 4.06–3.99 (m, 3H), 3.94 (d, *J* = 8.4 Hz, 1H), 3.83 (d, *J* = 6.8 Hz, 1H), 2.73–2.68 (m, 4H), 2.39 (d, *J* = 7.2 Hz, 2H), 2.35–2.28 (m, 1H), 2.25 (s, 3H), 2.10 (s, 3H), 2.06–1.95 (m, 2H), 1.89 (dq, *J* = 14.5, 7.5 Hz, 6H), 1.68 (s, 3H), 1.59–1.49 (m, 4H), 1.36 (s, 9H), 1.23 (s, 28H), 1.10 (s, 3H), 1.01 (s, 3H), 0.84 (d, *J* = 6.9 Hz, 3H).

### Preparation of Prodrug NPs

The LTX‐SS‐CA NPs were prepared by the one‐step nanoprecipitation method.^[^ ^10^, ^11^, ^31^ ^]^ Prodrugs and DSPE‐PEG_2k_ (weight ratio: 1:0.3) were both added into ethanol (2 mL). After they are totally dissolved, then added dropwise into deionized water (8 mL) with 800 rpm stirring. Then the rotary evaporator was utilized to remove the ethanol existed in the nanosystem. The characterizations (particle size, PDI, and zeta potential) of these NPs were detected through the Nano ZS Zetasizer instrument (Malvern, UK). The morphology of these LTX‐SS‐CA NPs was measured by the JEM‐2100 transmission electron microscope (TEM, JOEL, Japan).

### In Vitro Drug Release

The drug release was evaluated in two different mediums. Three kinds of NPs were incubated with rat plasma. And the LTX concentration was measured at the pre‐designed time points (0, 1, 2, 3, 4, 5, and 6 h). Then, the same kinds of NPs were also incubated in PBS (pH 7.4) and added to different concentrations of GSH (the molar ratio of prodrugs and GSH was 1:0, 1:1, 1:10, and 1:100). And the released LTX was measured at predetermined time points (0, 0.3, 0.5, 1, 2, 4, 6, 8, and 12 h). The released LTX concentration was calculated by the formula behind: LTX release rate (%) = Ct × *V*/*M* × 100 (Ct: the LTX concentration at the time point *t*; *V*: the released system volume; *M*: the amount of LTX in the nanosystem).

### Colloidal Stability

The average particle size and PDI were utilized to measure the colloidal stability. Briefly, *α* NPs, *β* NPs, and *γ* NPs (500 nmol mL^−1^) were incubated in PBS (pH 7.4) with 10% rat plasma (V V^−1^) at 37 °C. Meanwhile, the same NPs (500 nmol mL^−1^) were also incubated in PBS (10% rat plasma V V^–1^; pH7.4) with GSH (50 µmol mL^−1^) (the molar ratio of prodrug NPs and GSH is 1:100). At time points (0, 1, 2, 3, 4, 5, and 6 h), the mean particle size and PDI were measured and recorded.

### Cytotoxicity Assay

4T1 cells (1000 cells per well) were seeded into 96‐well plates and incubated for 24 h in a cell incubator (37 °C, 5% CO_2_). Then, serial dilutions of LTX solution, prodrug NPs, and prodrug NPs with GSH (molar ratio of NPs and GSH is 1:100) were added into pre‐designed wells and the cells in wells without any treatment were considered as the control. After incubation for 48 h, each well was washed with PBS three times, and MTT solution (5 mg mL^−1^) was added to the wells. After further 4 h incubation, DMSO was added to dissolve the purple crystal at the bottom of the wells, and absorbance at 570 nm was detected by a microplate reader (Model 500, USA). The IC_50_ was obtained through IBM SPSS Statistic 22.

### Cytotoxicity Uptake

4T1‐RFP cells and RAW264.7 cells were utilized to investigate the cellular uptake of LTX‐SS‐CA NPs with or without GSH. The 4T1‐RFP mixed with RAW264.7 cells group, 5 × 10^5^ 4T1‐RFP cells, and 5 × 10^5^ RAW264.7 cells were mixed and seeded in six‐well plates with 1 × 10^6^ cells per well. Then, 200 nmol mL^−1^ LTX solution or prodrug NPs were added to each well. For NPs + GSH groups, 20 µmol mL^−1^ GSH was added into each well with prodrugs NPs. After incubation for 2 or 8 h in a cell incubator (37 °C, 5% CO_2_), cells were transferred into EP tubes at a density of 1 × 10^6^ cells mL^−1^ for further cell‐sorting. For cell sorting, BD FACSArialIII (Becton, Dickinson and Company, New Jersey, USA) was used to sort cells in each well. Briefly, as for the size of 4T1‐RFP and RAW264.7 cells, an 85 µm nozzle was chosen for the sorting. According to the protocol of the manufacturer, 4T1‐RFP cells were stained by AF700 antibody and RAW264.7 cells were stained by AF700, CD45, CD11b, and F480. Then, because of the RFP in 4T1‐RFP cells, a PE‐CF594‐A fluorescence channel was chosen and the P6 gap was set up to sort 4T1‐RFP cells. And Q2 gap (F480^+^ and CD11b^+^) was set to sort RAW264.7 cells. For 4T1‐RFP mixed with RAW264.7 cells group, 20 × 10^4^ 4T1‐RFP cells were sorted from the P6 gap and 20 × 10^4^ RAW264.7 cells were sorted from the Q2 gap. The prodrug and LTX involved in these sorted cells were further analyzed through Waters Xevo TQ‐XS UPLC‐MS/MS (Waters Corporation, Milford, MA).

### Animal Studies

According to the *Guide for the Care and Use of Laboratory Animals*, all animal experiments were carried out and obtained the ethical approval of the Institutional Animal Ethical Care Committee (IAEC) from Shenyang Pharmaceutical University.

### Pharmacokinetic Study

Female 4T1 tumor‐bearing Balb/c mice (20–25 g) were utilized to investigate the pharmaceutic profiles of LTX‐SS‐CA NPs. When tumors grew about 150 mm,^[^ ^3^ ^]^ LTX solution (6 mg kg^−1^) and prodrug NPs (9 mg kg^−1^) were intravenously administrated into each group (*n* = 5). After the predetermined time points injection (2 min, 5 min, 20 min, 40 min, 1 h, 1.5 h, 2 h, 3 h, 4 h, 6 h, 8 h, 12 h, and 24 h), the mice were executed and their blood, tumors, hearts, livers spleens, lungs, and kidneys were collected. The prodrugs and LTX concentrations were measured through Waters Xevo TQ‐XS UPLC‐MS/MS (Waters Corporation, Milford, MA).

### In Vivo Antitumor Study

For the first in vivo antitumor efficacy experiments, 4T1 cells (5 × 10^6^) were injected subcutaneously into the right‐back of female Balb/c mice. When tumor volumes were ≈150 mm^3^, mice were intravenously injected with LTX solution (6 mg kg^−1^), saline, and NPs (9 mg kg^−1^) (*n* = 5). For NPs + GSH group, GSH (300 mg kg^−1^) was administrated immediately after NPs injection. As for NPs – GSH (2 h) group, GSH (300 mg kg^−1^) was injected after 2 h of NPs injection. There is a total of five injections every other day. And the body weight and tumor volume were measured every day. On day 12, mice were sacrificed and the blood was obtained for further hepatorenal functions analysis. In addition, the main organs and tumors of mice were utilized for H&E staining. The tumor slides were also used for Ki67 and TUNEL staining. For the second in vivo antitumor efficacy experiments, 4T1 tumor‐bearing mice were also established as the same method as the above. When tumor volume grew to ≈100 mm^3^, 300 mg kg^−1^ GSH was administrated to the mice of (GSH – Anti‐PD‐1) group, (GSH – *γ* NPs) group, (GSH – *γ* NPs – GSH (2 h)) group, and (GSH – *γ* NPs – GSH (2 h) + Anti‐PD‐1) group in five continuous days. For the other three groups, saline was injected into these mice on the same days. Then, Anti‐PD‐1 (40 µg/mice) and *γ* NPs (9 mg kg^−1^) were administrated into the mice in the corresponding group according to the pre‐designed dosing regimen on days 6, 8, 10, 12, 14, and 16. The tumor size and body weight were also recorded every day. After 3 d of the last dosage, mice were executed, and major organs, blood, and tumors were obtained for further analysis using the same experimental scheme as the first antitumor efficacy experiments. The tumor volume was calculated as the formula behind: Tumor volume (mm^3^) = Length × Width × Width × 0.5.

### Evaluation of GSH for ROS Inhibition

Different concentrations of GSH (6.25, 12.5, 25, and 50 µg mL^−1^) were utilized to evaluate the ROS inhibition effects on TGF‐*β* activated NIH3T3 cells. TGF‐*β* (10 ng mL^−1^) was added into NIH3T3 cells for 24 h and the cell concentration of each well was 1 × 10^3^. Then, different concentrations of GSH were added into different wells of groups (*n* = 5 for each group) and the cells were further incubated for 24 h. After that, the ROS concentration in NIH3T3 cells was measured through the Reactive Oxygen Species Assay Kit (Beyotime Biotechnology Ltd., Shanghai, China). And the fluorescence intensity was measured by BD FACS Celesta flow cytometry (Becton, Dickinson and Company, New Jersey, USA) through the BB‐515 fluorescence channel.

### MALDI‐MSI Analysis

The tumors were frozen in liquid nitrogen and sliced into 30 µm thick by cryomicrotome (Leica CM1950, Nussloch, Germany) at −20 °C. Then, slices were thawed on ITO glass slides. *α*‐Cyano‐4‐hydroxycinnamic acid (CHCA) matrix solution (20 mg mL^−1^, 1 mL) was added to the cavity of an airbrush (MR. Linear Compressor L7/PS270 Airbrush, Tokyo, Japan) to apply per ITO glass slide. The distance between the tip of the airbrush and the tissue surface was about 10 cm. After spraying, the solvent within slides was vaporized for MALDI‐MSI analysis. The operation parameters of MALDI‐MSI are as follows: mass range: m/z 800–1500 Da; ion polarity: positive; sample voltage: 3.50 kV; detector voltage: 1.90 kV; frequency: 1000 Hz; laser intensity: 25; irradiation diameter:10 µm; pitch: 70 µm × 70 µm. Then, 150 shots of each pixel were used to lighten the slides. And the data were processed by the MS Solution Version 1.30 software (Shimadzu Corporation, Tokyo, Japan).

### Immunofluorescence Staining

Tumor samples were soaked in 4% for 24 h at 4 °C. The tumors were embedded in optimal embedding medium through burying machine (Wuhan Junjie Electronics, Hubei, China) and cut into 10 µm sheets by Leica RM2016 cryostat (Shanghai Leica Instrument Co., Ltd., Shanghai, China). The slides were putted into the EDTA antigen retrieval buffer (pH 8.0) and washed three times with 1× PBS (pH 7.4). Then, samples were covered with 10% donkey serum at 25 °C for 30 min. After throwing away block solution, slides were incubated with the first primary antibodies overnight at 4 °C. Then, slides were washed three times with 1× PBS (pH 7.4) and incubated with secondary antibodies at 25 °C for 50 min in dark condition. After that, incubating slides with DAPI solution at room temperature for 10 min in dark place and then coverslip with anti‐fade mounting medium. Finally, slides were detected though Nikon Eclipse C1 Ortho‐Fluorescent microscopy (Nikon Co., Tokyo, Japan).

### Masson's Staining

Masson's staining was utilized to evaluate the amount of collagen among tumors. 4% PFA was used to fix the tumor tissues. After cutting tumors into slides, they were stained by Masson's staining kit (Wuhan Servicebio Technology Co., Ltd., HuBei, China). Then, the slides were observed by Nikon Eclipse C1 Ortho‐Fluorescent microscopy (Nikon Co., Tokyo, Japan) and five randomly selected microscopic fields were obtained and quantified by ImageJ software.

### Western Blot Analysis

Tumor issues were washed by cold PBS three times and a 100 mg tumor sample was homogenized with an electric homogenizer. Then, 1 mL RIPA buffer (Wuhan Servicebio Technology Co., Ltd., HuBei, China) was added into every sample. The total protein concentration was measured by the BCA protein concentration measurement kit (Wuhan Servicebio Technology Co., Ltd, HuBei, China). After that, 25 µg protein was utilized for western blot analysis.

### Immunohistochemistry

The tumor slides were placed in antigen retrieval buffer for 30 min. Then, the slides were placed in 3% hydrogen peroxide at 25 °C for 25 min under dark conditions. Subsequently, 3% BSA was added and these slides were sealed for 30 min at room temperature. After that, slides were incubated with primary antibody overnight at 4 °C, and then they were washed by PBS (pH 7.4) for three times. Then, these slides were covered with secondary antibodies from the corresponding species of primary antibody and incubated for 50 min at 25 °C.

### Flow Cytometry

The tumor issues were placed on the ice and incubated with collagenase A buffer and DNAase at 37 °C for 40 min. Then, FACS buffer was utilized to obtain a single‐cell suspension. After diluting the cell concentration to 1–2 × 10^6^ cells mL^−1^, they were stained with antibodies according to the protocol manufacturer. Finally, these samples were loaded into BD FACS Celesta flow cytometry (Becton, Dickinson and Company, New Jersey, USA) to measure and the results were analyzed by BD FACSDiVa software.

### Cytokines Analysis

Tumor samples were accurately weighed and saline was added to samples at the proportion of weight (mg):volume (UL) = 1:9. Then, these samples were mechanically homogenized and centrifugated for 10 min. The cytokines within supernatant were measured according to the instructions of the ELISA kit. Finally, the OD of each well was determined by a microplate reader (Model 500, USA).

### Statistical Analysis

The final data were expressed as means ± standard deviation (SD). The difference between two groups was calculated through the Student's *t*‐test. And the difference between multiple groups was analyzed by one‐way analysis of variance (ANOVA). GraphPad Prism 9.0 was utilized for the significance analysis. The *P<0.05, ** *P*<0.01, *** *P*<0.001, and **** *P*<0.0001 were considered significant.

## Conflict of Interest

The authors declare no conflict of interest.

## Author Contributions

S.D. and Y.Z. contributed equally to this work. S.D., Y.Z., Z.H., and Y.W. designed research; S.D., Y.Z., X.G., C.Z., Z.W., J.Y., Y.L., C.L., and Y.H. performed the research; S.D., Y.Z., B.S., M.S., H.Z., D.O., Z.H., and Y.W. analyzed the data; S.D. wrote the manuscript. Y.Z., Z.H., and Y.W. participated in the manuscript revision.

## Supporting information

Supporting Information

## Data Availability

The data that support the findings of this study are available in the supplementary material of this article.
