# Supplementary material for: Glutathione Pulse Therapy: Promote Spatiotemporal Delivery of Reduction‐Sensitive Nanoparticles at the “Cellular Level” and Synergize PD‐1 Blockade Therapy
Source: Adv Sci (Weinh). 2022 Jul 27;9(27):2202744. doi: 10.1002/advs.202202744 (PMC9507359; doi:10.1002/advs.202202744)
Supplement: Supplementary file 1 — Supporting Information [file ADVS-9-2202744-s001.pdf]

ADVANCED SCIENCE
Open Access

Supporting Information

for *Adv. Sci.*, DOI 10.1002/advs.202202744

Glutathione Pulse Therapy: Promote Spatiotemporal Delivery of Reduction-Sensitive Nanoparticles at the "Cellular Level" and Synergize PD-1 Blockade Therapy

Songtao Dong, Yuan Zhang, Xiangnan Guo, Chuang Zhang, Zhaomeng Wang, Jiang Yu, Yubo Liu, Chang Li, Yuting Hu, Bingjun Sun, Mengchi Sun, Haotian Zhang, Defang Ouyang, Zhonggui He and Yongjun Wang**

Supporting Information

Glutathione pulse therapy: promote spatiotemporal delivery of reduction-sensitive nanoparticles at the "cellular level" and synergize PD-1 blockade therapy

Songtao Dong[#], Yuan Zhang[#], Xiangnan Guo, Chuang Zhang, Zhaomeng Wang, Jiang Yu, Yubo Liu, Chang Li, Yuting Hu, Bingjun Sun, Mengchi Sun, Haotian Zhang, Defang Ouyang, Zhonggui He, Yongjun Wang**

This file includes:

Figures. S1 to S20
Tables. S1 to S3

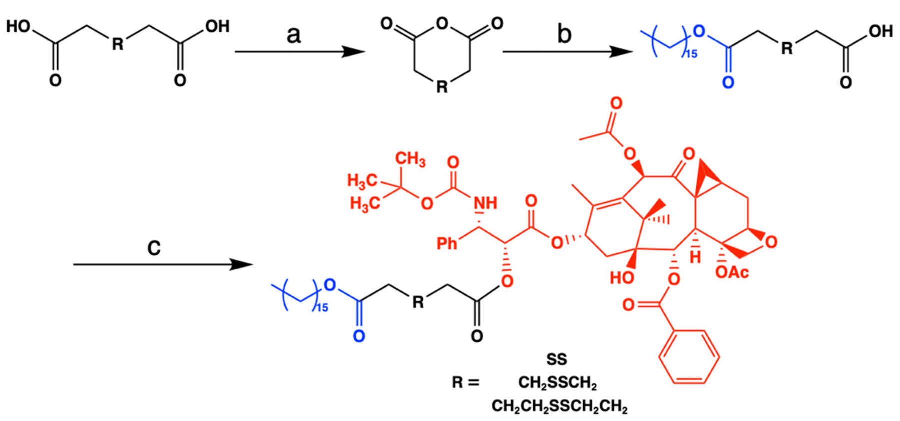

(a): acetic anhydride, 25°C; (b): Cetyl alcohol, DAMP, 25°C;

(c): EDCI, HoBt, DMAP, 0 °C; Larotaxel, 25°C

Figure S1. The synthetic route of the designed disulfide bond-bridged LTX-CA prodrugs.

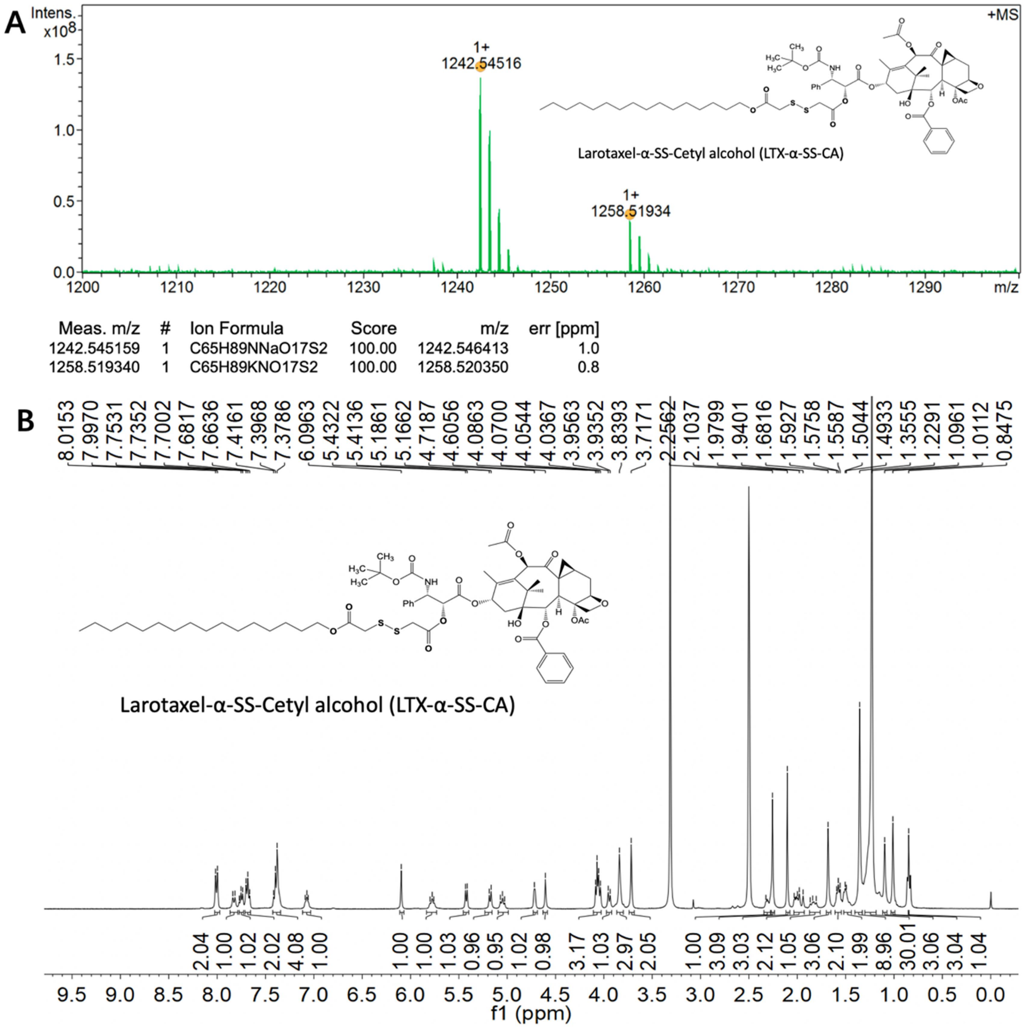

Meas. m/z	#	Ion Formula	Score	m/z	err [ppm]
1242.545159	1	C65H89NNaO17S2	100.00	1242.546413	1.0
1258.519340	1	C65H89KNO17S2	100.00	1258.520350	0.8

Figure S2. (**A**) MS and (**B**) ^{1}HMR of α LTX-SS-CA.

α LTX-SS-CA ($C_{65}H_{89}NO_{17}S_2$)

^{1}H NMR (400 MHz, DMSO-d_6) δ 8.01 (d, J = 7.3 Hz, 2H), 7.83 (d, J = 8.9 Hz, 1H), 7.77 – 7.73 (m, 1H), 7.68 (t, J = 7.3 Hz, 2H), 7.39 (d, J = 7.2 Hz, 4H), 7.07 (t, J = 6.6 Hz, 1H), 6.10 (s, 1H), 5.78 (t, J = 7.6 Hz, 1H), 5.42 (d, J = 7.5 Hz, 1H), 5.18 (d, J = 8.0 Hz, 1H), 5.05 (t, J = 8.4 Hz, 1H), 4.72 (s, 1H), 4.61 (s, 1H), 4.06 (q, J = 6.8 Hz, 3H), 3.95 (d, J = 8.4 Hz, 1H), 3.84 (s, 3H), 3.72 (s, 2H), 2.32 (s, 1H), 2.26 (s, 3H), 2.10 (s, 3H), 1.99 (dd, J = 22.1, 14.8 Hz, 2H), 1.87 – 1.76 (m, 1H), 1.68 (s, 3H), 1.61 – 1.55 (m, 2H), 1.50 (d, J = 4.4 Hz, 2H), 1.36 (s, 9H), 1.23 (s, 30H), 1.10 (s, 3H), 1.01 (s, 3H), 0.85 (s, 1H).

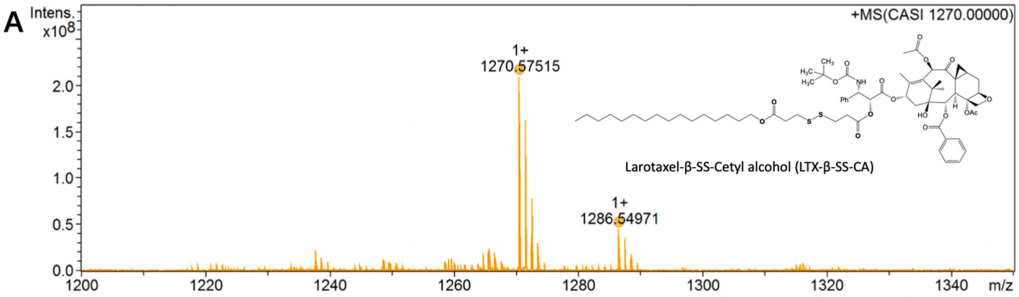

Meas. m/z	#	Ion Formula	Score	m/z	err [ppm]
1270.575149	1	C67H93NNaO17S2	100.00	1270.577713	2.0
1286.549711	1	C67H93KNO17S2	100.00	1286.551651	1.5

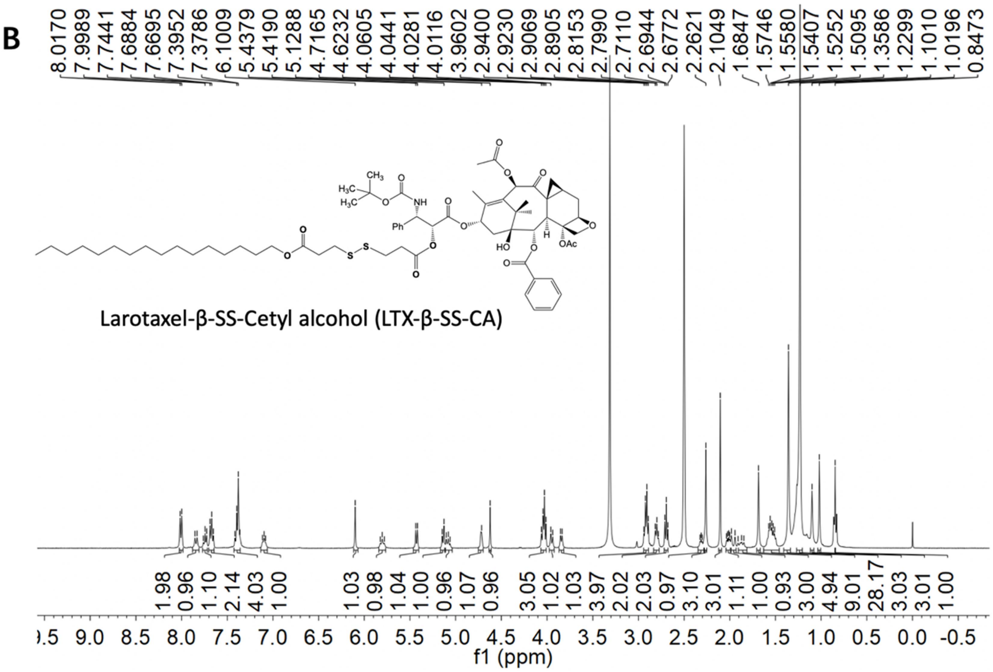

Figure S3. (**A**) MS and (**B**) ^{1}HMR of β LTX-SS-CA.

β LTX-SS-CA($C_{67}H_{93}NO_{17}S_2$)

^{1}H NMR (400 MHz, DMSO-d_6) δ 8.01 (d, J = 7.2 Hz, 2H), 7.84 (d, J = 9.3 Hz, 1H), 7.74 (t, J = 7.4 Hz, 1H), 7.67 (t, J = 7.4 Hz, 2H), 7.39 (q, J = 7.5, 7.0 Hz, 4H), 7.10 (t, J = 6.3 Hz, 1H), 6.10 (s, 1H), 5.80 (t, J = 9.1 Hz, 1H), 5.43 (d, J = 7.6 Hz, 1H), 5.14 (d, J = 7.6 Hz, 1H), 5.11 – 5.04 (m, 1H), 4.72 (s, 1H), 4.62 (s, 1H), 4.04 (q, J = 6.6 Hz, 3H), 3.95 (d, J = 8.5 Hz, 1H), 3.84 (d, J = 7.3 Hz, 1H), 2.92 (q, J = 6.7 Hz, 4H), 2.80 (t, J = 6.5 Hz, 2H), 2.69 (t, J = 6.8 Hz, 2H), 2.35 – 2.28 (m, 1H), 2.26 (s, 3H), 2.10 (s, 3H), 2.02 (dd, J = 10.0, 4.6 Hz, 1H), 1.96 (d, J = 16.0 Hz, 1H), 1.86 (d, J = 9.9 Hz, 1H), 1.68 (s, 3H), 1.54 (dt, J = 12.5, 6.5 Hz, 5H), 1.36 (s, 9H), 1.23 (s, 28H), 1.10 (s, 3H), 1.02 (s, 3H), 0.85 (s, 1H).

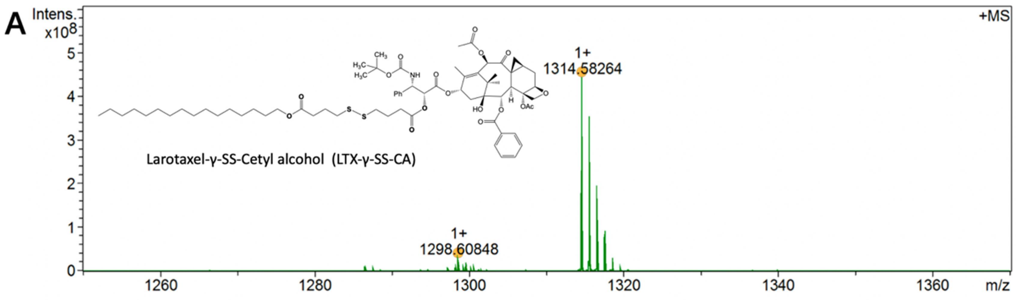

Meas. m/z	#	Ion Formula	Score	m/z	err [ppm]
1298.608477	1	C69H97NNaO17S2	100.00	1298.609013	0.4
1314.582643	1	C69H97KNO17S2	100.00	1314.582951	0.2

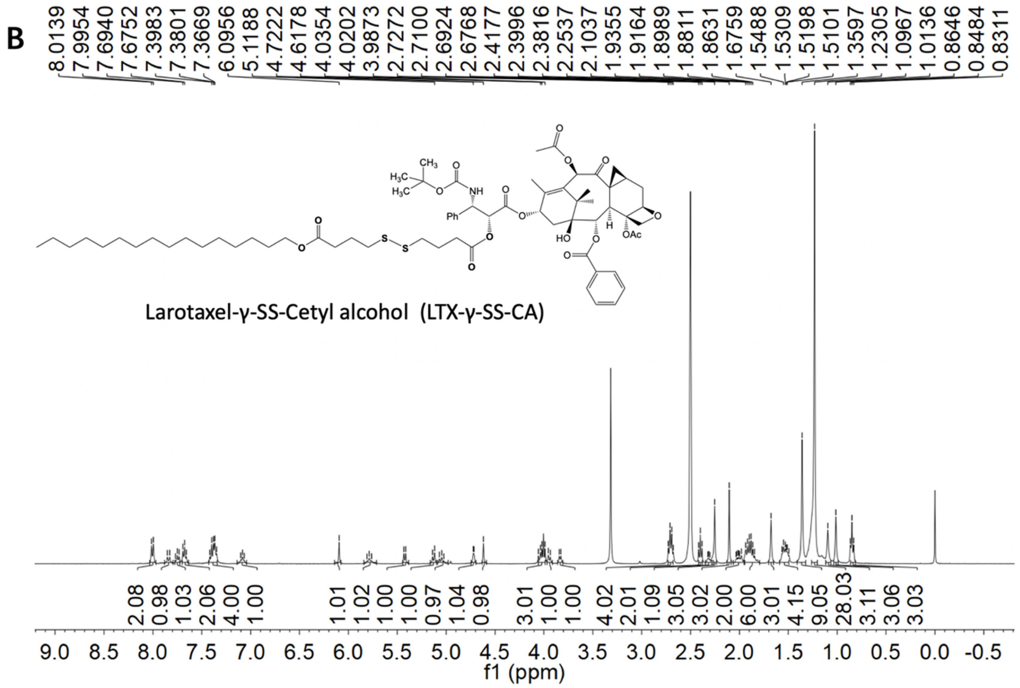

Figure S4. (**A**) MS and (**B**) ^{1}HMR of γ LTX-SS-CA.

γ LTX-SS-CA ($C_{69}H_{97}NO_{17}S_2$)

^{1}H NMR (400 MHz, DMSO-d_6) δ 8.00 (d, *J* = 7.4 Hz, 2H), 7.84 (d, *J* = 9.4 Hz, 1H), 7.75 (t, *J* = 7.5 Hz, 1H), 7.68 (t, *J* = 7.4 Hz, 2H), 7.38 (dt, *J* = 12.4, 7.4 Hz, 4H), 7.08 (t, *J* = 7.2 Hz, 1H), 6.10 (s, 1H), 5.79 (t, *J* = 9.3 Hz, 1H), 5.44 – 5.38 (m, 1H), 5.13 (d, *J* = 8.0 Hz, 1H), 5.05 (t, *J* = 8.9 Hz, 1H), 4.72 (d, *J* = 3.0 Hz, 1H), 4.62 (s, 1H), 4.06 – 3.99 (m, 3H), 3.94 (d, *J* = 8.4 Hz, 1H), 3.83 (d, *J* = 6.8 Hz, 1H), 2.73 – 2.68 (m, 4H), 2.39 (d, *J* = 7.2 Hz, 2H), 2.35 – 2.28 (m, 1H), 2.25 (s, 3H), 2.10 (s, 3H), 2.06 – 1.95 (m, 2H), 1.89 (dq, *J* = 14.5, 7.5 Hz, 6H), 1.68 (s, 3H), 1.59 – 1.49 (m, 4H), 1.36 (s, 9H), 1.23 (s, 28H), 1.10 (s, 3H), 1.01 (s, 3H), 0.84 (d, *J* = 6.9 Hz, 3H).

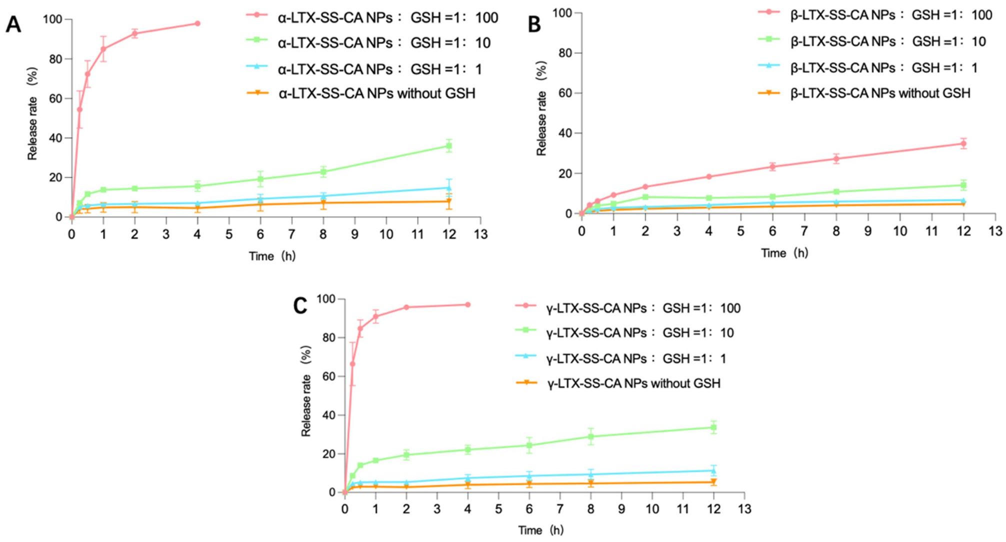

Figure S5. *In vitro* reduction-responsive drug release of prodrug nanoassemblies in the presence of various concentrations of GSH (n=3). (**A**) α LTX-SS-CA; (**B**) β LTX-SS-CA; (**C**) γ LTX-SS-CA.

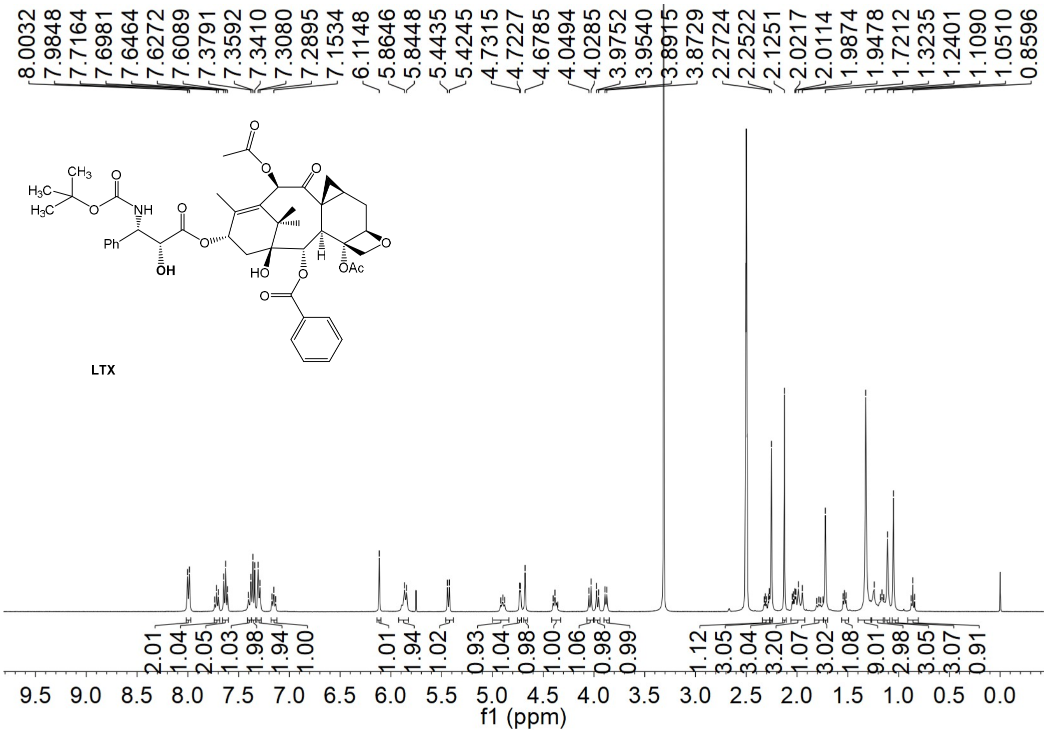

Figure S6. ^{1}HMR of LTX.

LTX ($C_{45}H_{53}NO_{14}$)

^{1}H NMR (400 MHz, DMSO-d_6) δ 7.99 (d, J = 7.4 Hz, 2H), 7.72 (t, J = 7.3 Hz, 1H), 7.63 (t, J = 7.5 Hz, 2H), 7.39 (d, J = 9.6 Hz, 1H), 7.35 (d, J = 7.3 Hz, 2H), 7.30 (d, J = 7.4 Hz, 2H), 7.15 (t, J = 7.2 Hz, 1H), 6.11 (s, 1H), 5.85 (d, J = 7.9 Hz, 2H), 5.43 (d, J = 7.6 Hz, 1H), 5.00 – 4.84 (m, 1H), 4.73 (d, J = 3.5 Hz, 1H), 4.68 (s, 1H), 4.39 (d, J = 7.1 Hz, 1H), 4.04 (d, J = 8.4 Hz, 1H), 3.96 (d, J = 8.5 Hz, 1H), 3.88 (d, J = 7.4 Hz, 1H), 2.34 – 2.27 (m, 1H), 2.25 (s, 3H), 2.13 (s, 3H), 2.06 – 1.92 (m, 3H), 1.78 (dd, J = 15.7, 9.1 Hz, 1H), 1.72 (s, 3H), 1.56 – 1.49 (m, 1H), 1.32 (s, 9H), 1.27 – 1.15 (m, 3H), 1.11 (s, 3H), 1.05 (s, 3H), 0.91 – 0.81 (m, 1H).

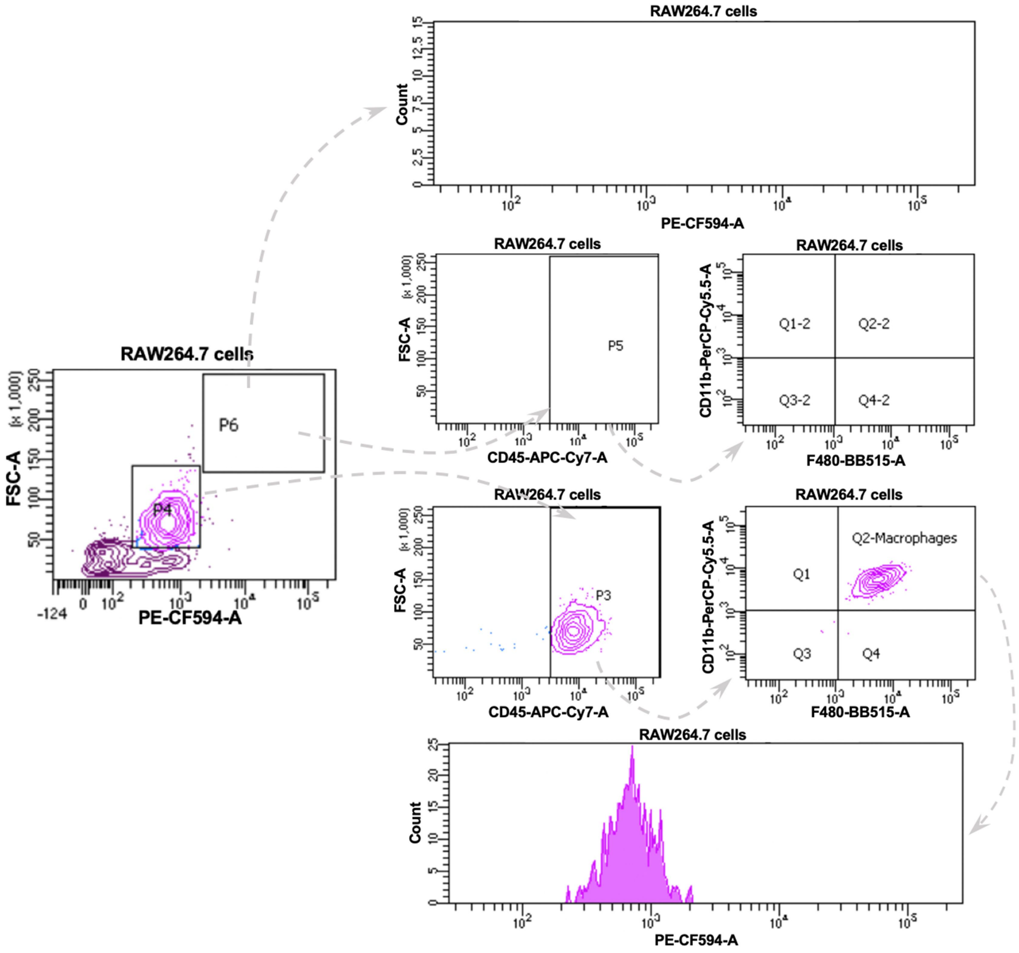

Figure S7. FACS method for RAW264.7 cells.

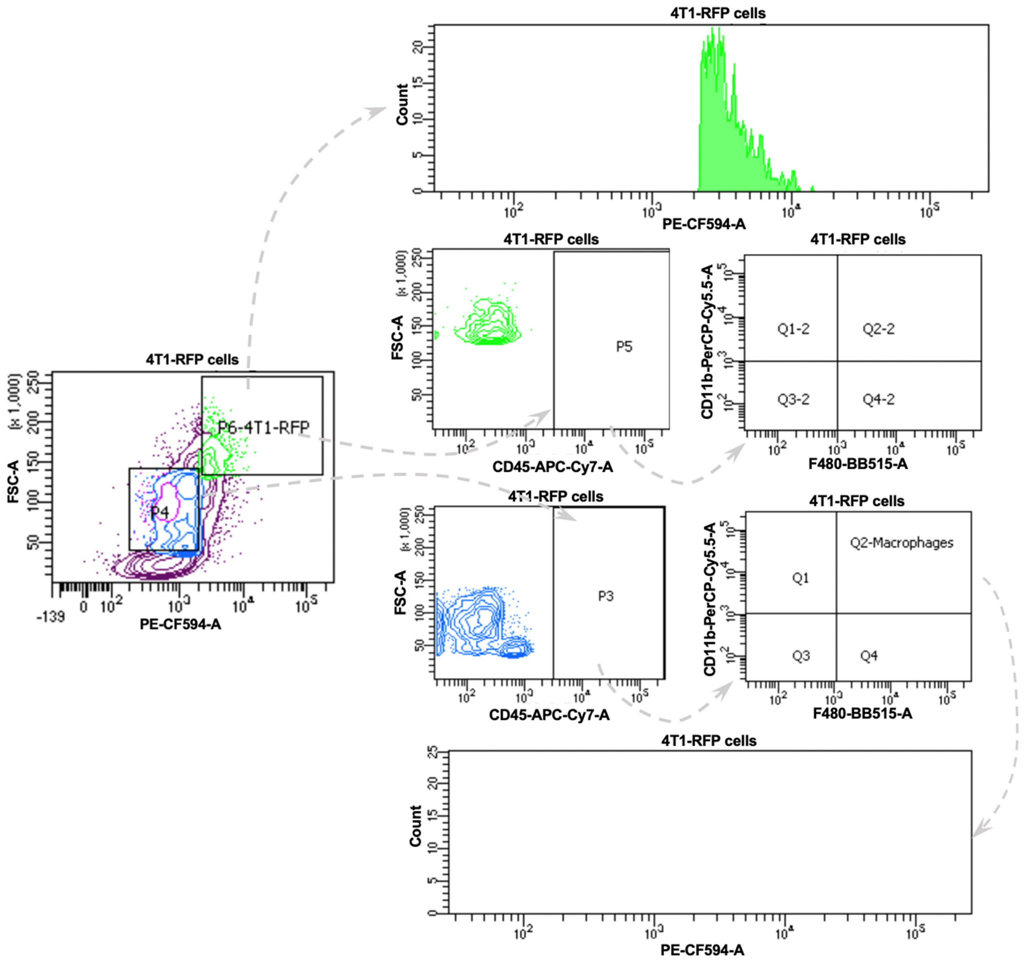

Figure S8. FACS method for 4T1-RFP cells.

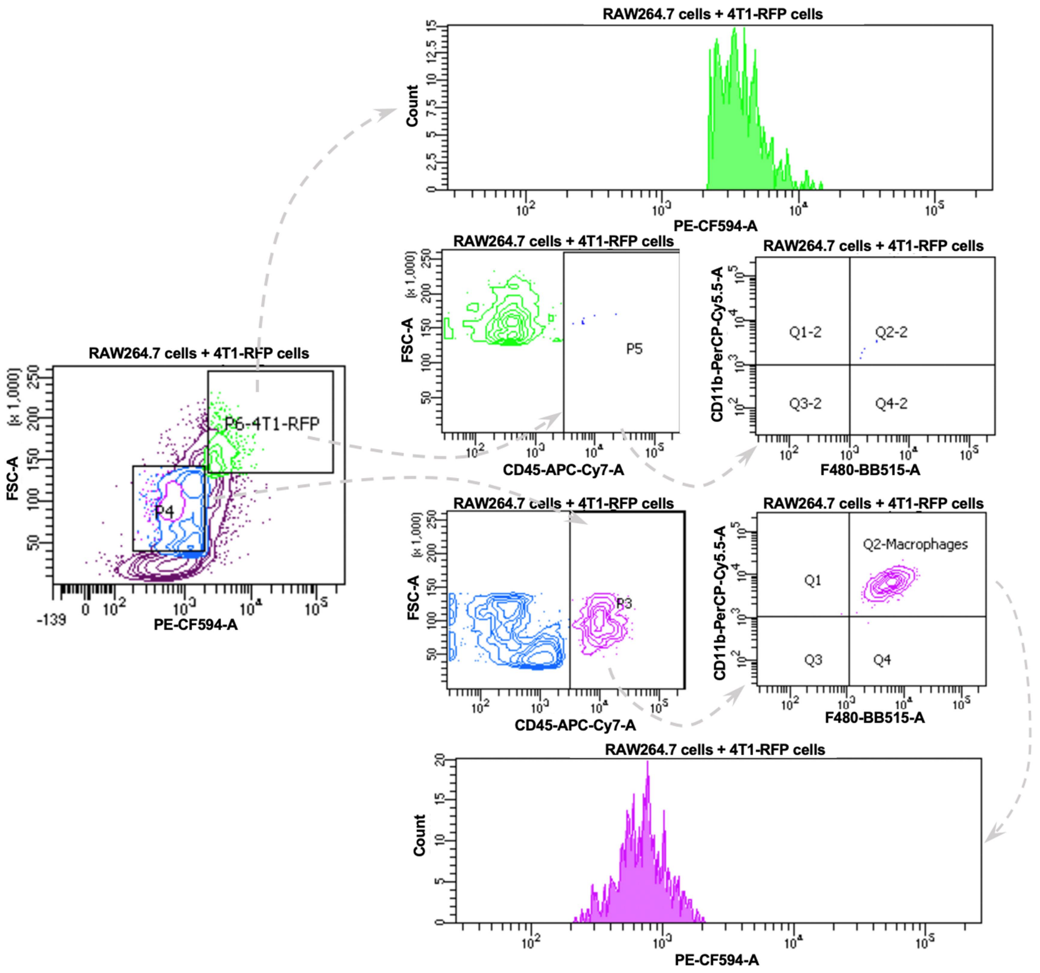

Figure S9. FACS method for 4T1-RFP cells mixed with RAW264.7 cells.

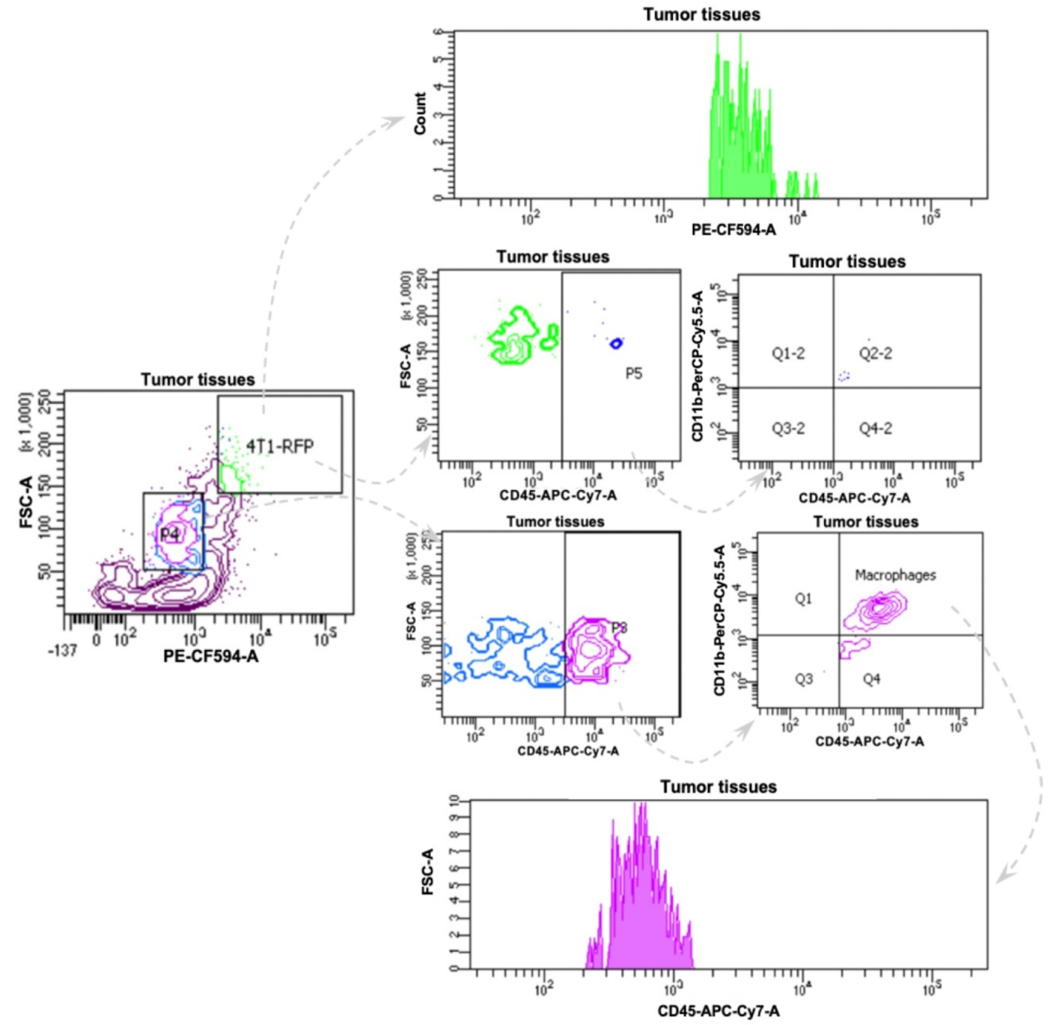

Figure S10. FACS method for tumor tissues.

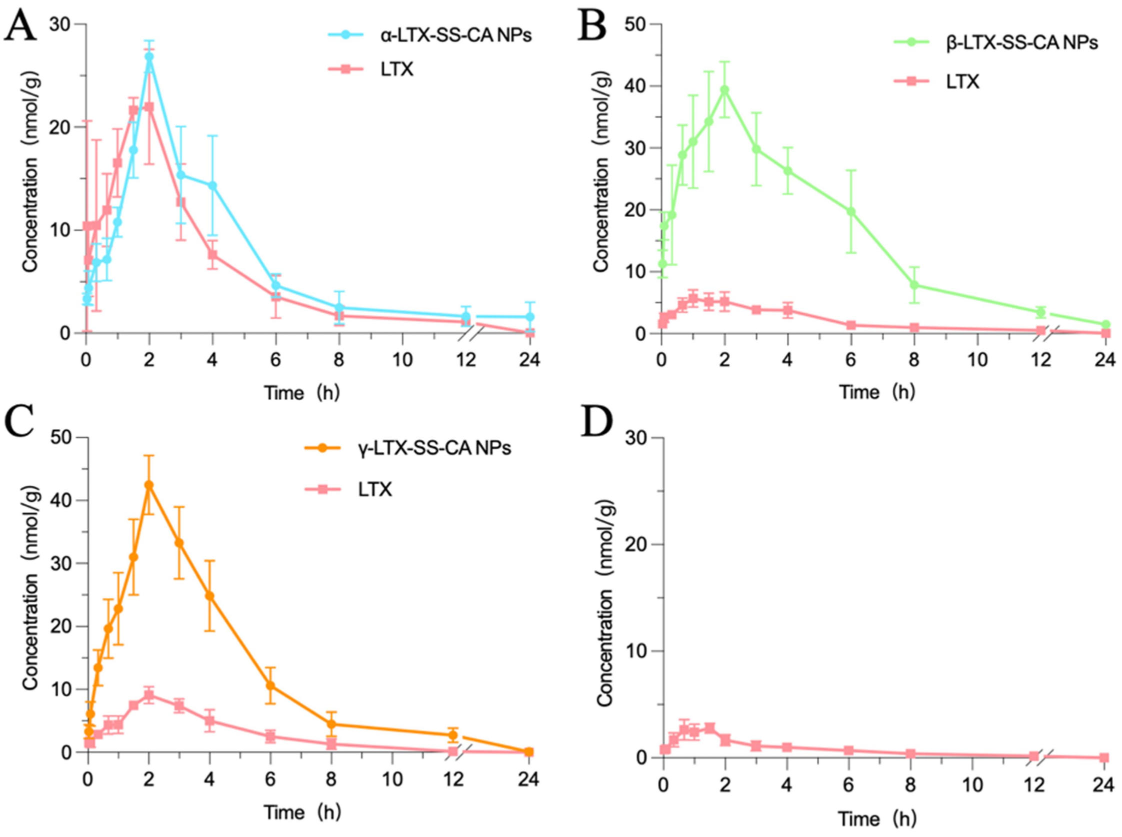

Figure S11. Tumor concentration-time profiles of α (**A**), β (**B**), γ (**C**) prodrugs and LTX solution (**D**) in Balb/c mice after intravenous injection of LTX solution or LTX-SS-CA prodrug NPs (7.2 μmol/kg) (n=5).

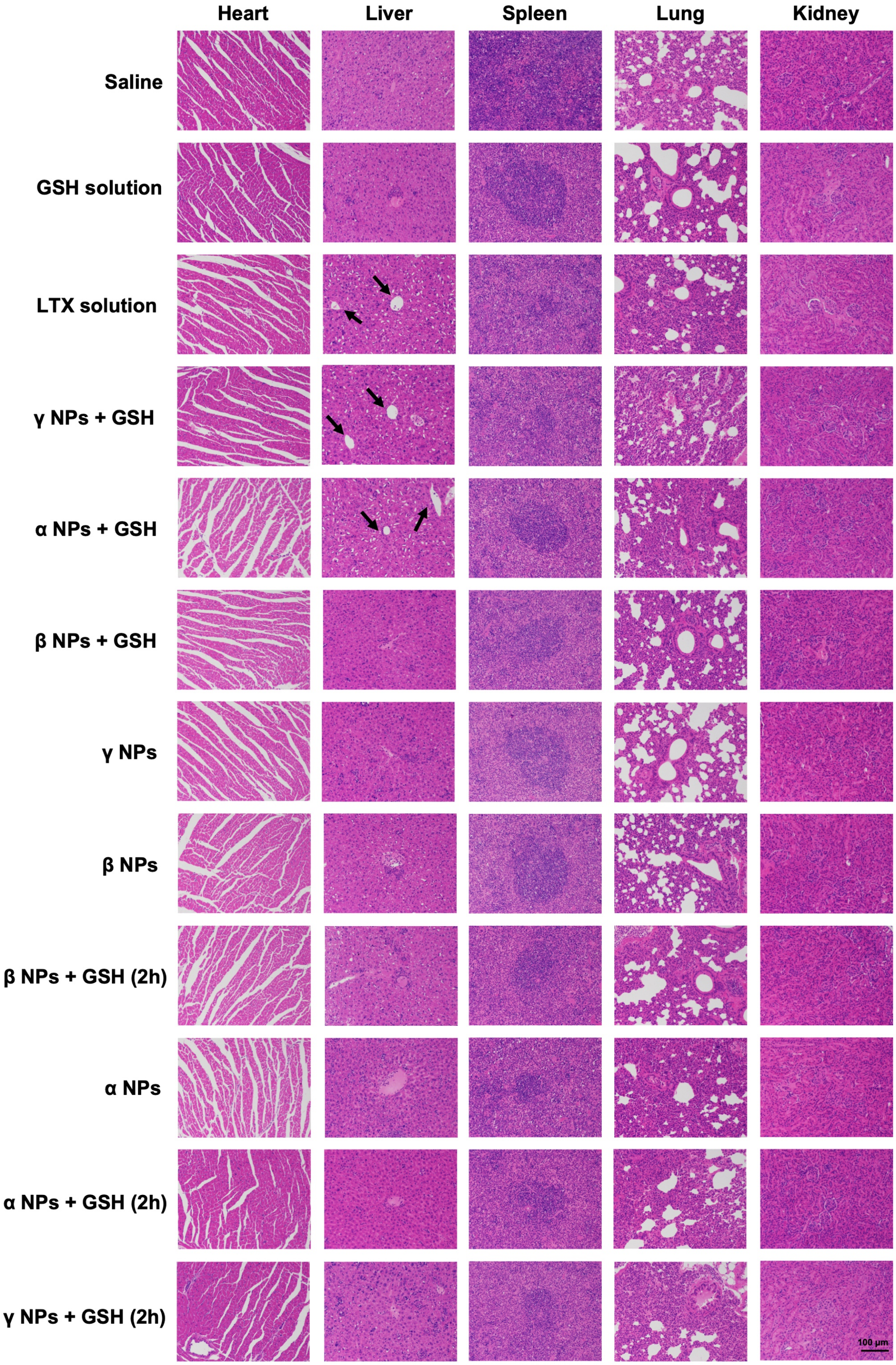

Figure S12. H&E staining of major organs and tumors of mice bearing 4T1 tumor xenografts after treatments.

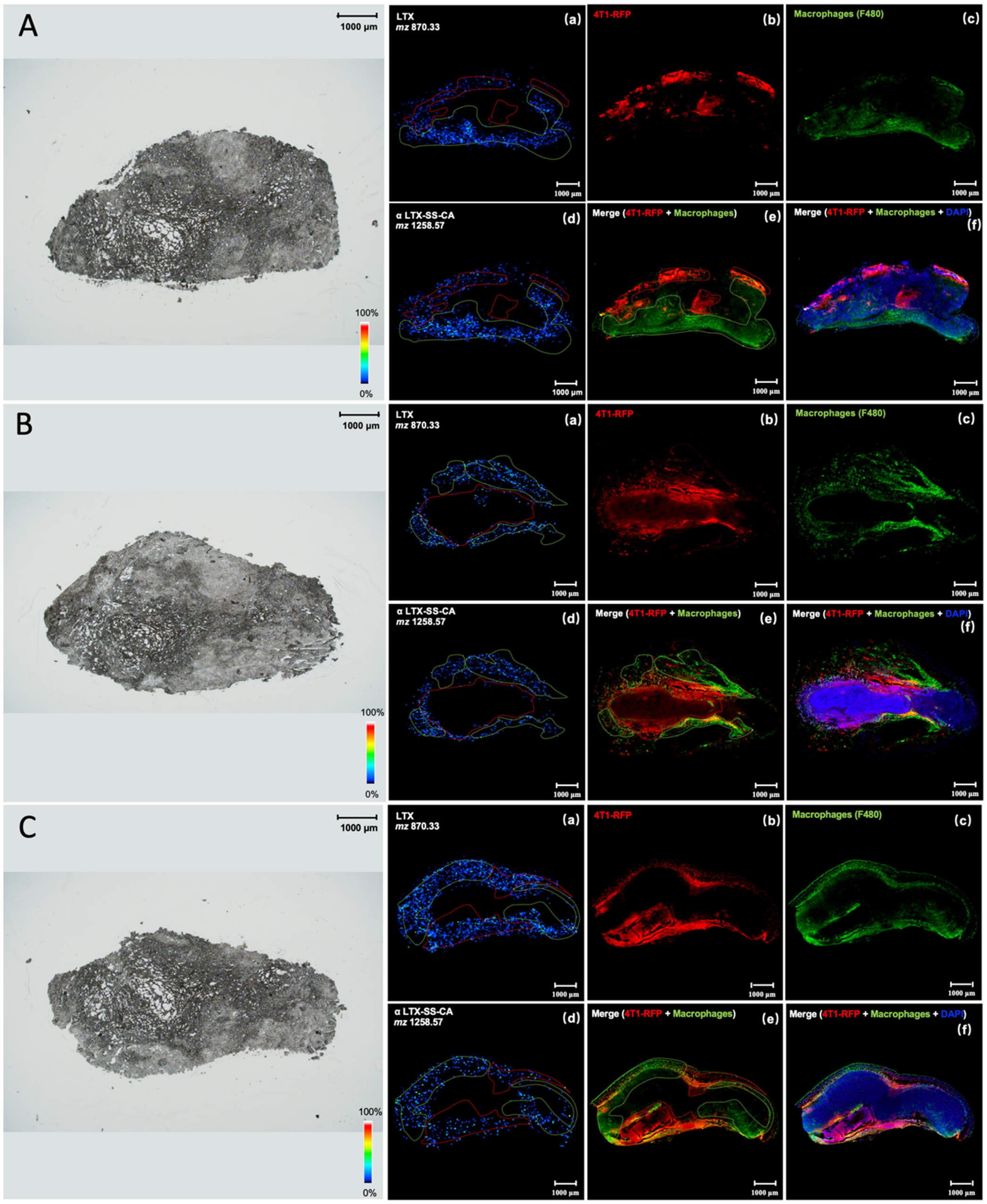

Figure S13. The optical images, PFOA-related MALDI signal ([M + K^+] at m/z 870.33 for LTX; [M + K^+] at m/z 1258.57 for α LTX-SS-CA prodrug) and fluorescence images (RFP, red; F480, green; DAPI, blue) of tumor tissues from tumor-bearing mice after treated with α NPs (**A**), α NPs + GSH (**B**), and α NPs + GSH (2h) (**C**).

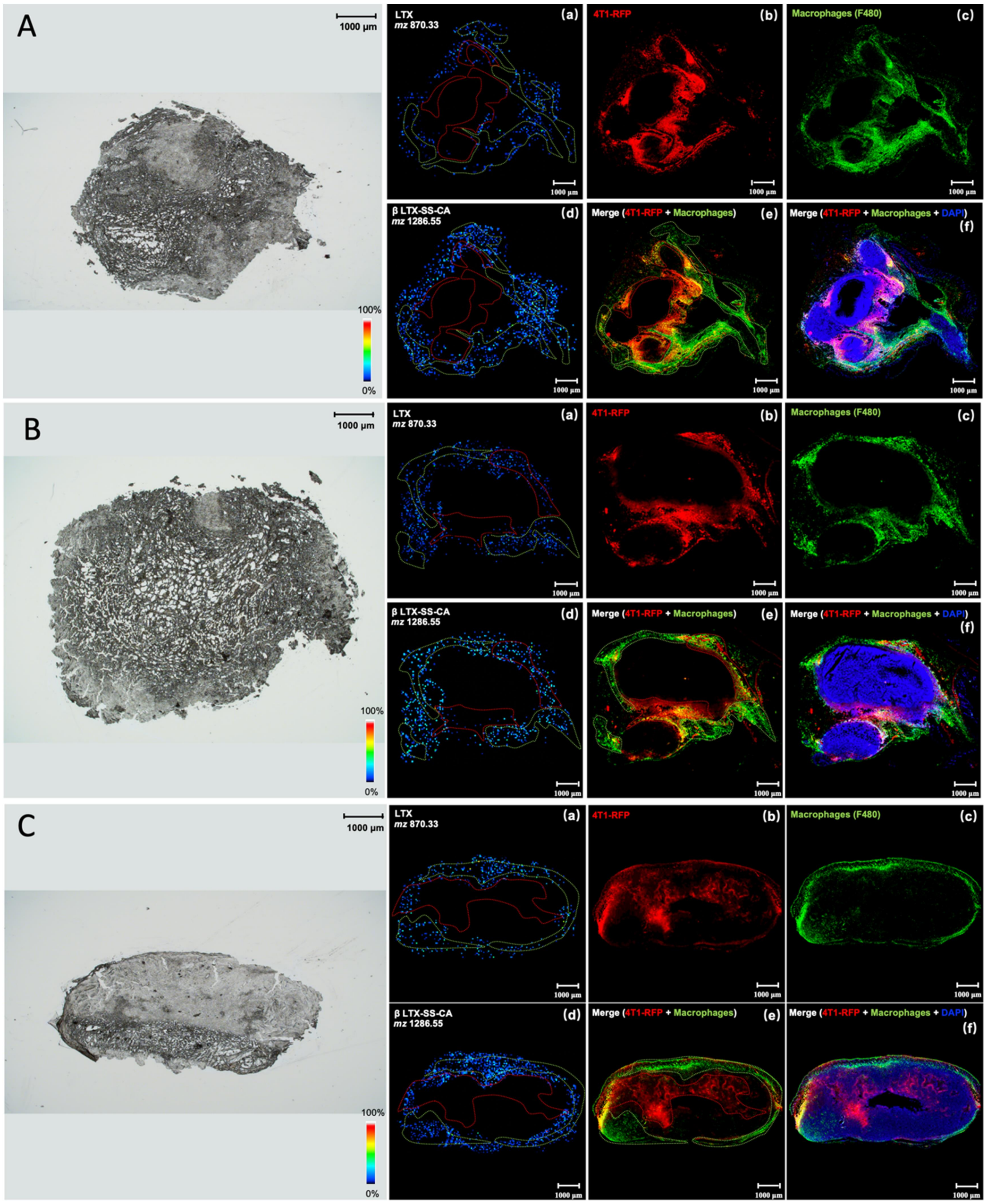

Figure S14. The optical images, PFOA-related MALDI signal ([M + K^+] at m/z 870.33 for LTX; [M + K^+] at m/z 1286.55 for β LTX-SS-CA prodrug) and fluorescence images (RFP, red; F480, green; DAPI, blue) of tumor tissues from tumor-bearing mice after treated with β NPs (**A**), β NPs + GSH (**B**), and β NPs + GSH (2h) (**C**).

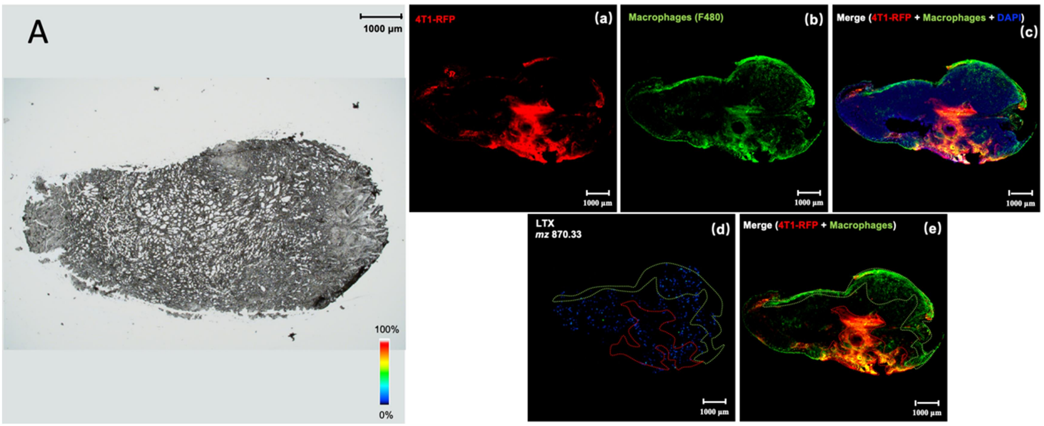

Figure S15. The optical images, PFOA-related MALDI signal ([M + K^+] at m/z 870.33 for LTX) and fluorescence images (RFP, red; F480, green; DAPI, blue) of tumor tissues from tumor-bearing mice after treated with LTX solution.

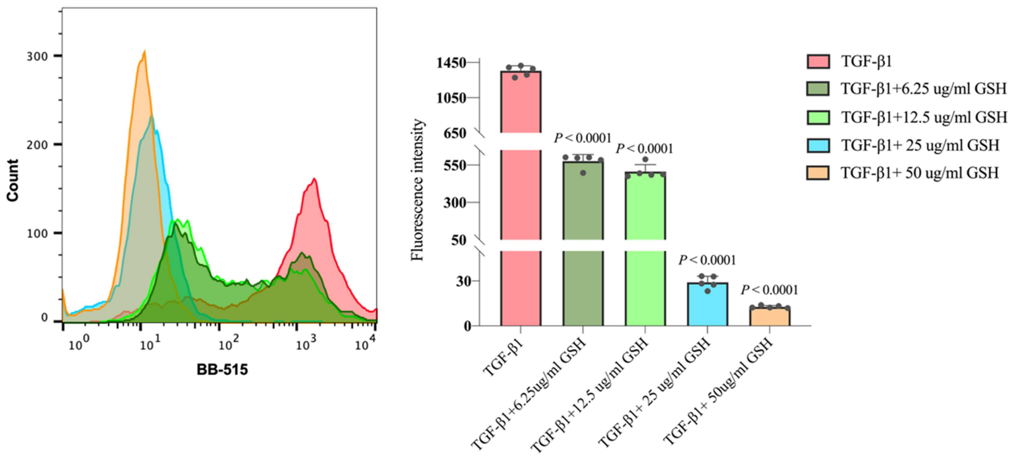

Figure S16. Effects of GSH on ROS inhibition in TGF-β (10 ng/mL) activated NIH3T3 cells.

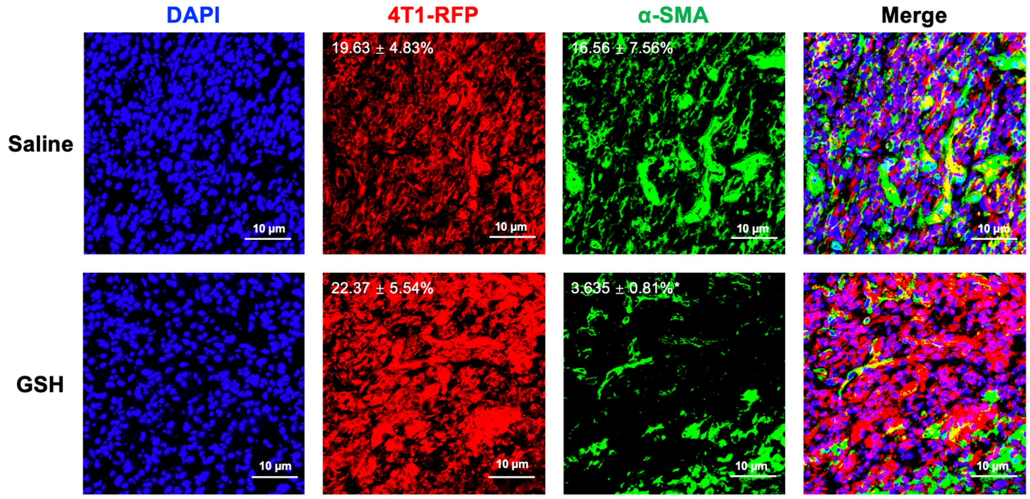

Figure S17. Confocal microscopy identifying α-SMA. The quantification results expressed as the percentage of total cell number (n=5). Statistical significance: n.s.= not significant, *$P<$ 0.05, ** $P<0.01$, *** $P<0.001$, and **** $P<0.0001$.

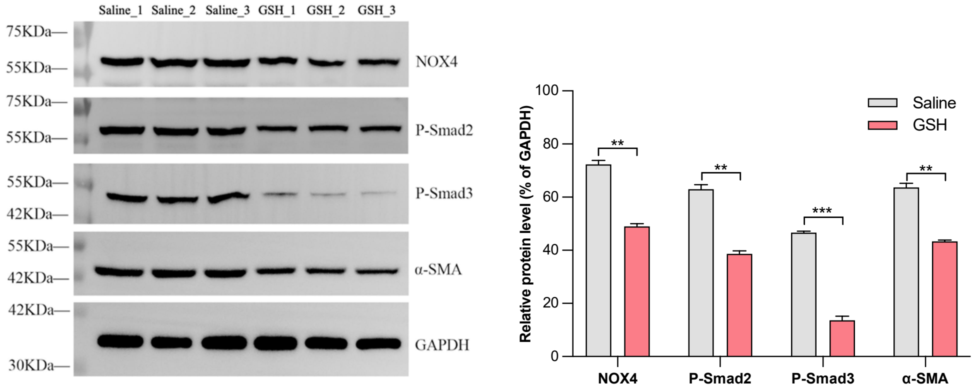

Figure S18. Western blot analysis of NOX4, P-Smad2, P-Smad3, α-SMA and GAPDH expression in the 4T1 tumor after GSH treatments (n=3). Protein expression levels were quantified by ImageJ, and normalized with GAPDH. Statistical significance: n.s.= not significant, $*P < 0.05$, $** P < 0.01$, $*** P < 0.001$, and $**** P < 0.0001$.

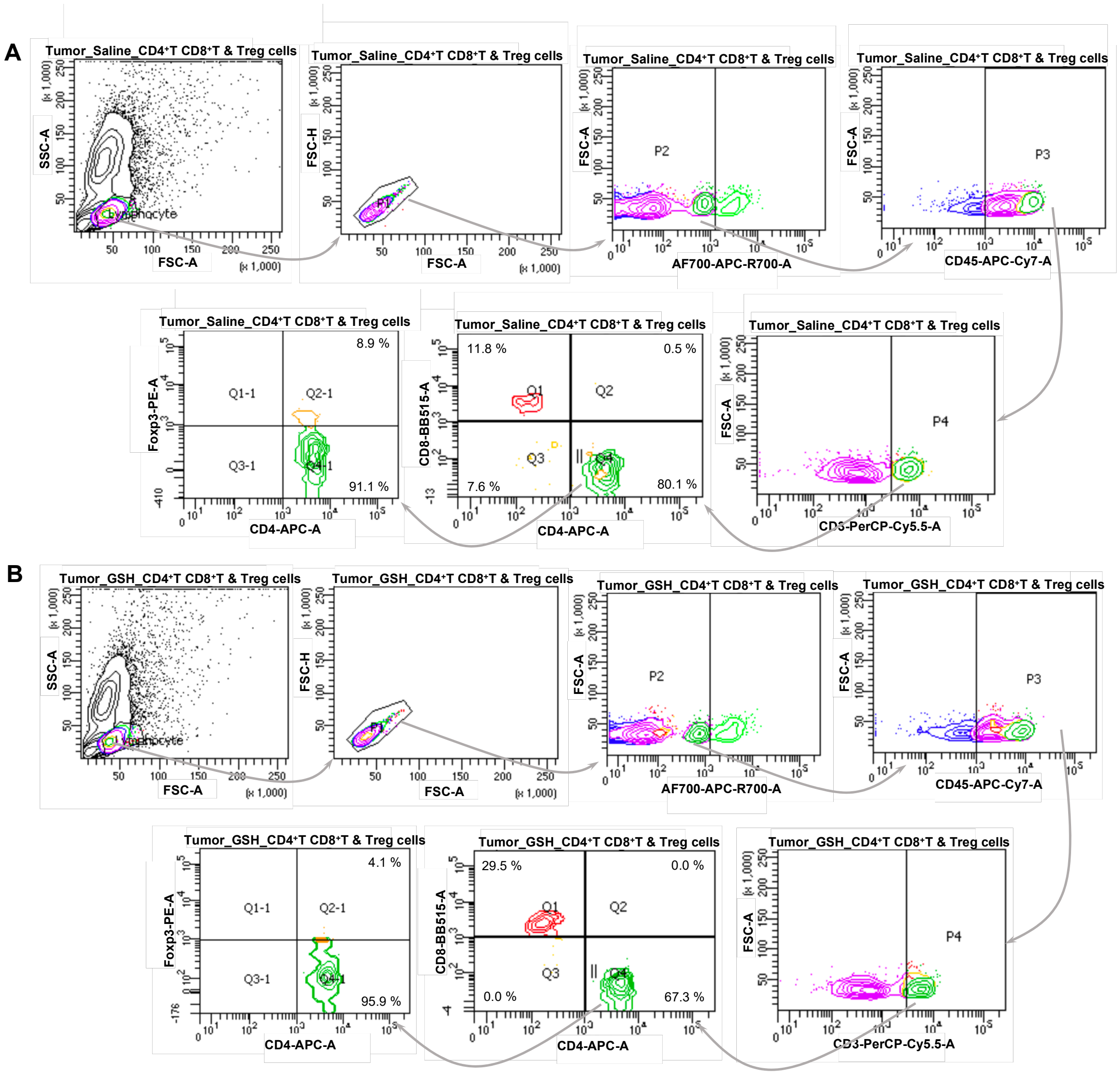

Figure S19. Cells analysis method and gating strategies of flow cytometry for $CD4^+$ T cells, $CD8^+$ T cells and Tregs of tumor tissues obtained from tumor bearing mice after treated with saline and GSH.

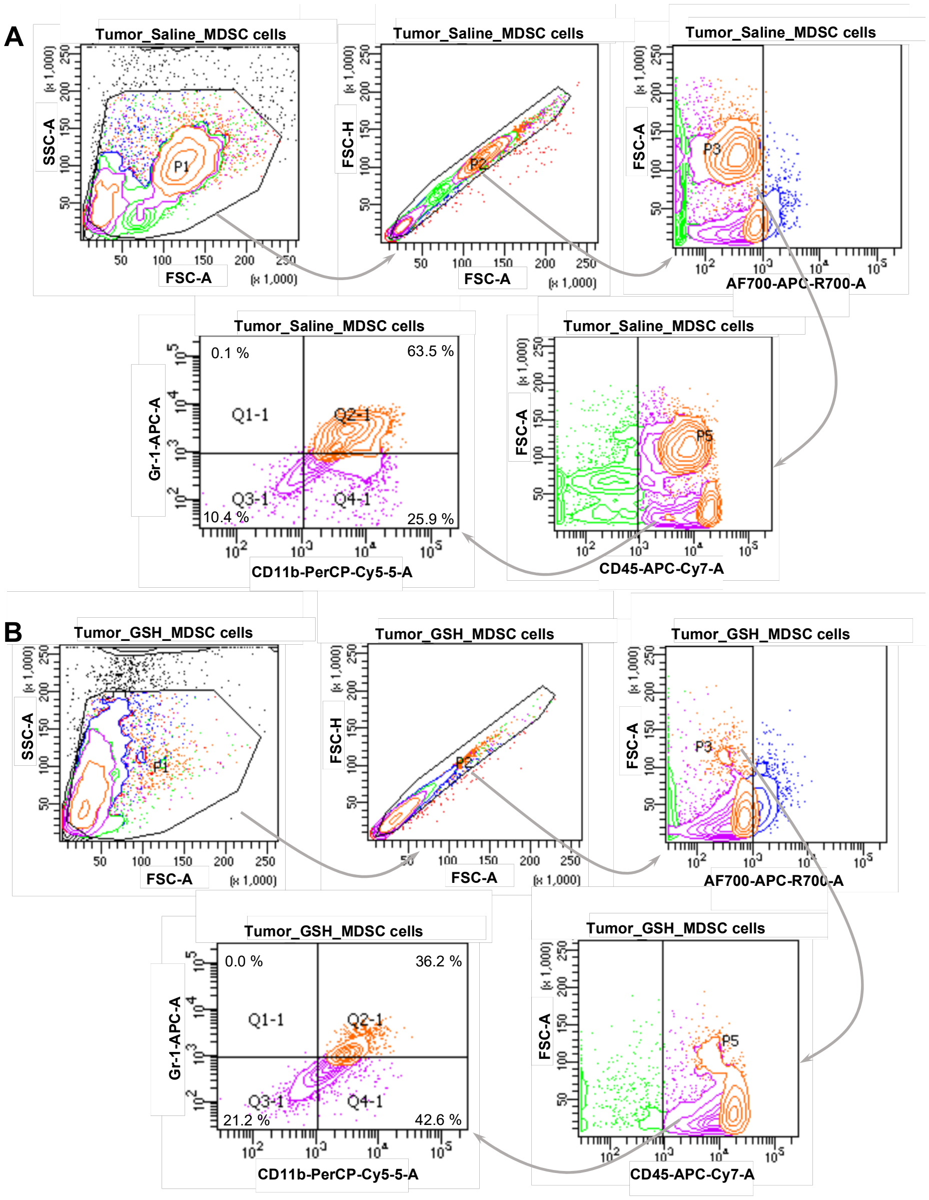

Figure S20. Cells analysis method and gating strategies of flow cytometry for MDSC cells of tumor tissues obtained from tumor bearing mice after treated with saline and GSH.

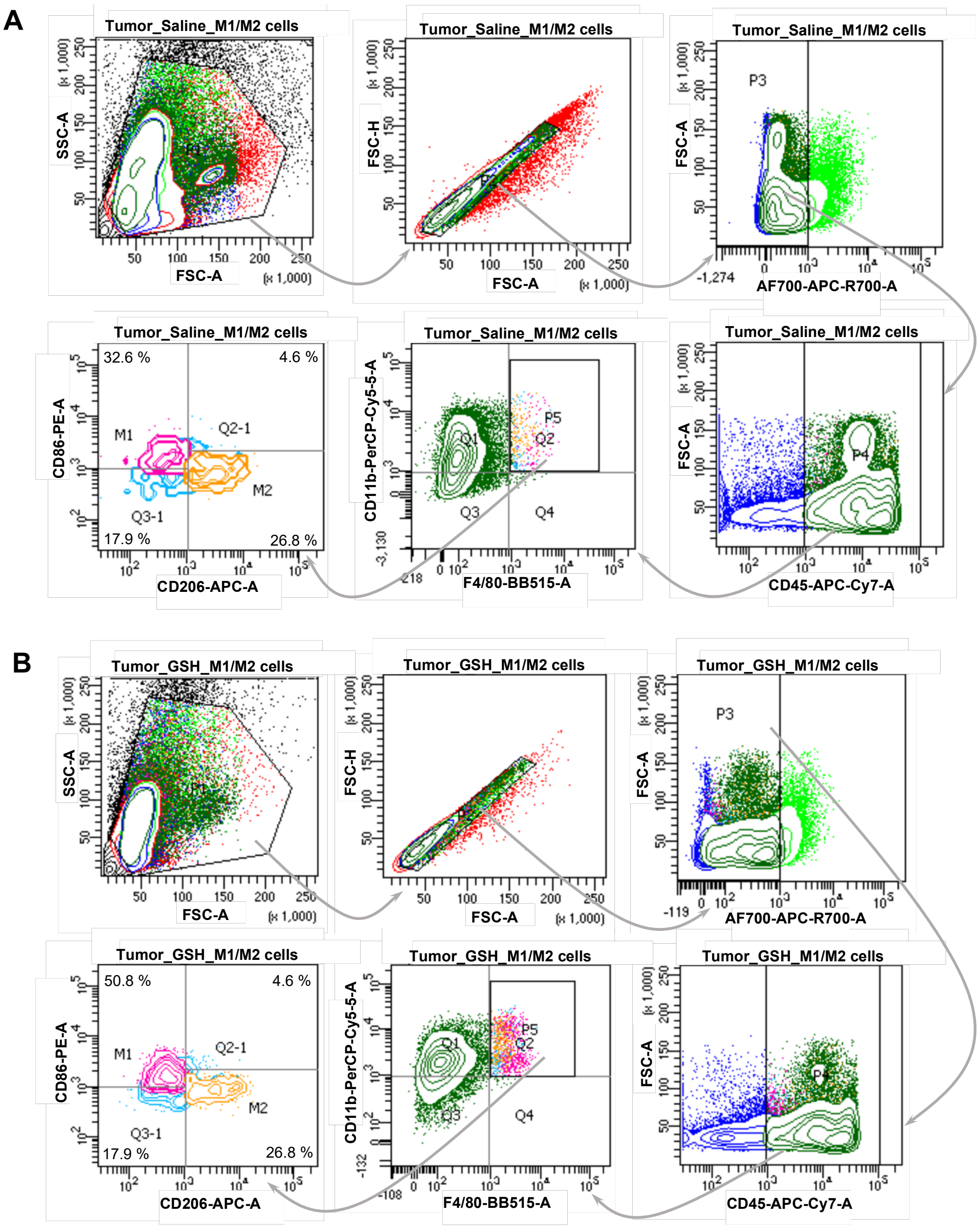

Figure S21. Cells analysis method and gating strategies of flow cytometry for the ratio of M1 cells / M2 cells within tumor tissues obtained from tumor bearing mice after treated with saline and GSH.

Table S1. Characteristics of nanoassemblies (n=3)

Nanoassemblies	Size [d.nm][a)]	PDI[a)]	Zeta potential [mV][a)]	LTX drug loading [%][b)]
α LTX-SS-CA NPs	108 ± 2.4	0.13 ± 0.02	-29.5 ± 0.8	59.5
β LTX-SS-CA NPs	100 ± 2.7	0.09 ± 0.01	-27.3 ± 0.9	57.8
γ LTX-SS-CA NPs	105 ± 2.5	0.11 ± 0.02	-28.1 ± 0.7	56.4

[a)] Mean diameters, polydispersity index, and zeta potential of nanoassemblies were determined by DLS; [b)] Drug loading of LTX was calculated by the molecular weight of conjugates and the amount of DSPE-PEG2k.

Table S2. Tumor pharmacokinetic parameters of LTX and prodrug NPs (n=5).

Formulations	Determined[a)]	AUC (nmol/ (g· L) · h)[b)]	T_{max}(h)[c)]	$t_{1/2}$ (h)[d)]	C_{max} (nmol/L)[e)]
LTX Solution	LTX	11.36 ± 1.71	0.83	1.90 ± 0.53	2.64 ± 0.95
α LTX-SS-CA NPs	LTX	87.13 ± 5.44[C]	2	2.62 ± 0.59	21.65 ± 1.21[C]
	α LTX-SS-CA	115.02 ± 10.40	2	2.57 ± 1.31	26.85 ± 1.56
β LTX-SS-CA NPs	LTX	30.76 ± 2.65[CF]	1	3.57 ± 2.22	5.68 ± 1.37[BF]
	β LTX-SS-CA	247.09 ± 19.6[F]	2	3.75 ± 1.86	39.43 ± 4.51[F]
γ LTX-SS-CA NPs	LTX	39.73 ± 6.36[CFG]	2	1.92 ± 0.51	9.10 ± 1.35[CFH]
	γ LTX-SS-CA	196.02 ± 10.51[FP]	2	2.50 ± 0.43	42.46 ± 1.35[F]

a) Prodrugs and the released LTX were simultaneously determined. **b**) Area under the plasma concentration-time curve. **c**) Peak time. **d**) Half-time. **e**) Peak concentration. (**h**) A: $P < 0.05$, B: $P < 0.01$, C: $P < 0.001$, versus LTX solution as the control. D: $P < 0.05$, E: $P < 0.01$, F: $P < 0.001$, versus α LTX-SS-CA NPs as the control. G: $P < 0.05$, H: $P < 0.01$, I: $P < 0.001$, versus β LTX-SS-CA NPs as the control (Two-tailed student's t-test). Data are presented as mean ± SD.

Table S3. Plasma pharmacokinetic parameters of LTX and prodrug NPs (n=5).

Formulations	Determined[a)]	AUC (nmol/L · h)[b)]	$t_{1/2}$ (h)[c)]	Cl(L/h/kg)[d)]	Vd(L/kg)[e)]
LTX Solution	LTX	7.63 ± 2.53	7.66 ± 2.99	1.11 ± 0.27	12.16 ± 5.62
α LTX-SS-CA NPs	LTX	17.42 ± 0.88^{C}	6.77 ± 2.99	0.46 ± 0.03 ^{C}	4.40 ± 1.76^{A}
	α LTX-SS-CA	28.35 ± 3.25	4.55 ± 1.57	0.28 ± 0.02	1.86 ± 0.67
β LTX-SS-CA NPs	LTX	8.72 ± 1.36^{F}	8.28 ± 3.84	0.96 ± 0.17 ^{F}	10.98 ± 3.51
	β LTX-SS-CA	53.48 ± 3.85^{C}	4.46 ± 0.95	0.16 ± 0.01^{F}	1.018 ± 0.19^{A}
γ LTX-SS-CA NPs	LTX	10.2 ± 0.85^{F}	7.73 ± 2.63	0.82 ± 0.07^{G}	9.17 ± 3.29
	γ LTX-SS-CA	67.65 ± 2.42^{CP}	5.2 ± 1.28	0.13 ± 0.01^{FG}	0.95 ± 0.24^{A}

a) Prodrugs and the released LTX were simultaneously determined. **b**) Area under the plasma concentration-time curve. **c**) Half-time. **d**) Clearance. (**e**) Apparent volume of distribution. (**f**) A: $P < 0.05$, B: $P < 0.01$, C: $P < 0.001$, versus LTX solution as the control. D: $P < 0.05$, E: $P < 0.01$, F: $P < 0.001$, versus α LTX-SS-CA NPs as the control. G: $P < 0.05$, H: $P < 0.01$, I: $P < 0.001$, versus β LTX-SS-CA NPs as the control (Two-tailed student's t-test). Data are presented as mean ± SD.
